# Gene Expression Alterations Associated With Resveratrol‐Induced Antiproliferative Effects and S‐Phase Cell Cycle Arrest in Osteosarcoma Cancer Cells

**DOI:** 10.1111/jcmm.71111

**Published:** 2026-04-06

**Authors:** Raffaella De Pace, Maria Rosa Iaquinta, Roberta Chiarelli, Maria Giulia Dell'Aquila, Fabio Casciano, Cinzia Brenna, Elisa Mazzoni

**Affiliations:** ^1^ Department of Translational Medicine University of Ferrara Ferrara Italy; ^2^ Department of Medical Sciences University of Ferrara Ferrara Italy; ^3^ University Center for Studies on Gender Medicine, University of Ferrara Ferrara Italy; ^4^ Department of Environmental and Prevention Sciences and LTTA Centre University of Ferrara Ferrara Italy; ^5^ Laboratory for Technologies of Advanced Therapies “LTTA”‐Electron Microscopy Center University of Ferrara Ferrara Italy

**Keywords:** apoptosis, epithelial–mesenchymal transition (EMT), osteosarcoma (OS), resveratrol (RSV), Wnt/β‐catenin

## Abstract

Osteosarcoma (OS) is an aggressive primary bone tumour with high metastatic potential. Current treatments including surgery and chemotherapy are limited by side effects and chemoresistance, underscoring the need for novel therapies. This study aimed to investigate the antitumor potential of resveratrol (RSV), a natural polyphenol, as a novel treatment for OS. The effects of RSV were evaluated in two osteosarcoma cell lines (SAOS‐2 and U2‐OS). A viability assay established 100 μM as the effective concentration, and hyperspectral imaging confirmed cellular uptake. Apoptosis was measured via caspase‐3/7 activity and Annexin V/PI staining, while qRT‐PCR assessed pro‐apoptotic gene expression. Flow cytometry evaluated cell‐cycle progression, and a wound‐healing assay measured migration. Gene expression analyses (qRT‐PCR) examined markers of cell adhesion, tumour progression and epithelial–mesenchymal transition. Finally, RSV's impact on the Wnt/β‐catenin pathway was determined by quantifying nuclear β‐catenin accumulation and the expression of its downstream oncogenic targets. RSV inhibited cell proliferation and induced apoptosis, increasing caspase‐3/7 activity and modulating apoptotic gene expression. RSV also caused cell cycle arrest in S‐phase. It reduced the cells' migration and altered the expression of cell adhesion and tumour progression genes, promoting a less invasive phenotype. Notably, RSV decreased nuclear β‐catenin accumulation, downregulated oncogenic targets like c‐Myc and MMPs, and upregulated E‐cadherin while reducing vimentin levels, suggesting a reversal of epithelial–mesenchymal transition. These results suggest that RSV may offer a promising therapeutic approach for osteosarcoma, modulating key pathways involved in tumour progression, metastasis and chemoresistance. Further studies are required to assess its clinical applicability.

## 1 Introduction

Osteosarcoma (OS) is the most common malignant bone tumour in adolescents and children [1, 2]. With the original locations mostly in the proximal tibia and distal femur, osteosarcoma is the most frequent primary malignant bone tumour in children and adolescents [3]. Today surgery and supplemental multidrug chemotherapy are the main treatments for osteosarcoma; patients have a 77% 5‐year survival rate after this [4]. OS has a bimodal age distribution, peaking between 10–30 and 70–80 years, with the latter often linked to pre‐existing bone diseases or radiation exposure [5]. The primary symptoms include localised pain, swelling and restricted joint movement, with pathological fractures sometimes being the first sign of disease [6]. Metastasis remains a major obstacle limiting the survival of patients with osteosarcoma. Poor response to neoadjuvant chemotherapy and metastatic disease at diagnosis were confirmed as primary risk factors of poor outcome [7]. Cross‐talk between cancer‐associated fibroblasts (CAFs) and OS cells has been found to facilitate metastasis [8, 9]. The primary treatment for OS includes surgical tumour resection and chemotherapy with agents like methotrexate (MTX), cisplatin (DDP), doxorubicin (DOX) and ifosfamide (IFO), with etoposide used in metastatic cases [6, 10]. The most limiting factors include complications and fatal toxicity associated with chemotherapy agents [11, 12]. As a result of poorly characterised oncogenic events that arise in osteogenic lineage precursors, osteosarcoma (OS) develops in this complicated environment. There is growing evidence that the bone microenvironment (BME) is the driving force for OS onset and progression, much like it is in normal development [13]. Therefore, new therapeutic strategies are needed to improve efficacy and reduce side effects. Natural products have long been an important research area for the discovery of novel and bioactive molecules. Resveratrol (RSV), a natural polyphenol with antioxidant, anti‐inflammatory and antitumor properties, has emerged as a promising candidate [14]. Preclinical studies have shown that RSV has anticancer effects in various malignancies, including colorectal, lung, liver, breast, prostate and pancreatic cancers, demonstrating synergy with chemotherapy [15, 16]. RSV acts at multiple stages of carcinogenesis—initiation, promotion, progression and metastasis—while enhancing chemotherapy efficacy and reducing side effects [17, 18]. Recent data report that at the molecular level, RSV exerts antitumor effects by suppressing oncogenic proteins, activating caspases and inhibiting epithelial–mesenchymal transition (EMT) [19]. Resveratrol can affect both healthy and tumour cells, but its impact is context‐dependent. In healthy cells, it generally exerts protective effects by activating antioxidant defences, reducing oxidative stress and modulating anti‐inflammatory pathways. In contrast, tumour cells, which often exhibit elevated basal oxidative stress and rely on oncogenic signalling for survival, are more susceptible to resveratrol‐induced cytotoxicity. In these cells, resveratrol further increases reactive oxygen species (ROS) levels beyond the tolerance threshold, inhibits prosurvival pathways such as Wnt/β‐catenin and c‐Myc and activates apoptotic signalling, thereby selectively limiting tumour growth and invasiveness [20, 21, 22, 23]. Additionally, RSV modulates the balance of apoptotic proteins in the Bcl‐2 family, reducing antiapoptotic proteins while increasing proapoptotic factors, leading to caspase activation and DNA fragmentation in cancer cells [24]. The aetiology of OS is multifactorial, with 30% of cases associated with hereditary genetic mutations or syndromes [25] and 70% linked to acquired factors such as ionising radiation and previous chemotherapy [26]. Alterations in mesenchymal stem cells or osteoblastic progenitors impair osteogenic differentiation, leading to uncontrolled proliferation and tumour formation [27]. Key molecular changes include the silencing of tumour suppressor genes p53 and Rb, overexpression of the proto‐oncogene c‐Myc, and dysregulation of the Wnt/β‐catenin signalling pathway, all contributing to tumour progression, metastasis, and poor prognosis [28, 29, 30]. Epithelial–mesenchymal transition (EMT) plays a crucial role in OS metastasis by enhancing cell migration and invasiveness [31, 32]. During EMT, epithelial markers like E‐cadherin are lost, while mesenchymal markers such as N‐cadherin and vimentin are upregulated [33, 34, 35]. In OS, EMT is mediated by the Wnt/β‐catenin pathway, which promotes tumour progression through the overexpression of metalloproteinases (MMPs), degrading the extracellular matrix (ECM) [36, 37]. Normally, β‐catenin is degraded in the cytoplasm unless Wnt signalling is active, allowing it to translocate into the nucleus and drive promoting gene expression [38]. In OS, Wnt/β‐catenin signalling is often hyperactive due to mutations in its regulatory components, leading to uncontrolled proliferation and metastasis [38, 39]. Targeting this pathway presents a potential therapeutic strategy [40, 41]. This study evaluates the effects of RSV on OS cell lines SAOS‐2 and U2‐OS, focusing on its impact on cell proliferation, apoptosis, migration and EMT‐related gene expression.

## 2 Materials and Methods

### 2.1 Cell Culture and Chemicals

In vitro assays were conducted using OS cell lines SAOS‐2 and U2‐OS (ATCC, Cat. no. HTB‐85; Cat. no. HTB‐96) [42], along with human bone marrow‐derived mesenchymal stem cells (hBMSCs) (Lonza Milan, Italy, PT‐2501). hBMSCs were characterised by flow cytometry analysis (FCA) for MSC surface markers, including positive markers (CD29, CD73 and CD90) and negative markers (CD14 and CD45) [43]. Cells were maintained at 37°C in a humidified atmosphere with 5% CO_₂_ in DMEM/F12 medium (Euroclone, Milan, Italy), supplemented with 10% FBS (Cat. no. ECS0180L, Euroclone, Milan, Italy) and 2% penicillin/streptomycin (P/S) (Lonza, Milan, Italy). Cells were grown to 80% confluence in monolayer culture before being treated with RSV for 24, 48, and 72 h. Resveratrol (Merck, Milan, Italy, Cat. no. Y0001194) was prepared in double‐distilled water at concentrations of 1–1000 μM.

### 2.2 Hyperspectral Imaging (HSI)

To analyse the entry and localisation of the RSV compound within OS cells, a hyperspectral imaging analysis was performed (*n* = 3). OS cell (5 × 10^4^/well) were seeded on a glass slide, treated with RSV 100 μM for 48 h and then fixed with 4% PFA, covered and imaged. Dark‐field images were acquired using an Olympus microscope equipped with a CytoViva (CytoViva, Auburn, AL) dark‐field illumination system. The setup included a CytoViva dark‐field condenser, a 60× oil immersion objective (Olympus, UPLFLN100XOI2–2 Plan Fluorite, N.A. 0.6–1.3), and a 150 W quartz halogen light source (Dolan Jenner DC‐950, Massachusetts, USA). The hyperspectral camera spanned the 400–1000 nm spectrum. The post‐process imaging was performed by ENVI, the proprietary software of the instrument. To image resveratrol, a solution of 100 μM was made. Then, 10 μL of solution were loaded on a glass slide, let dry and covered with a coverslip. Acquisition parameters were: magnification, 60×; resolution 1024*1024; exposure time 0.15 s, lightsource power 75%. Post‐imaging, a resveratrol hyperspectral spectral library was developed. Twenty‐five single manually chosen pixels were recorded, encompassing the complete optical projection of resveratrol, making an endmembers' collection. Then, the Spectral Angle Mapper (SAM) algorithm was applied to generate a spatially resolved colour map, using the resveratrol spectral library previously built, as a reference library. The pixel percentage occupied by resveratrol spectra in control and treated cells was calculated by ENVI.

### 2.3 Cell Viability Assay

The MTT (3‐(4,5‐dimethylthiazol‐2‐yl)‐2,5‐diphenyl tetrazolium bromide) assay (Sigma‐Aldrich, Merk Life Science, Milan, Italy, Cat. no. M5655) was used to assess cell viability in OS cells and hBMSCs (*n* = 3). The experiment and subsequent analysis were carried out following the methods of Lanzilotti et al. [42]. Cells (5 × 10^4^/well) were seeded in 96‐well plates and treated with RSV (1–1000 μM) for 24, 48 and 72 h. MTT solution was added to each well at a final concentration of 0.5 μg/mL. The viable cells contain NAD(P)H‐dependent oxidoreductase enzymes which reduce the MTT to formazan. After a 4‐h incubation at 37°C, purple formazan crystals were solubilised with dimethyl sulfoxide (DMSO) and absorbance was measured at 570 nm using a spectrophotometer (Thermo Electron Corporation, Multiskan EX, Finland). The cell viability was expressed as a percentage relative to the untreated cells [42].

Live/Dead Cell Double Staining Kit (Merk, Milan, Italy, Cat. no. QIA76) was employed to analyse the viability of cells (*n* = 3). The experiment and subsequent analysis were carried out following the methods of Lanzilotti et al. [42]. OS cells (5 × 10^4^/well) were plated in 24‐well plates and treated with RSV 100 μM for 48 h at 37°C in a humidified atmosphere with 5% CO_2_. Cell‐permeable green fluorescent Cyto‐dye (Ex. max.: 488 nm; Em. max.: 518 nm) was used to stain live cells, whereas red fluorescent propidium iodide (PI) (Ex. max.: 488 nm; Em. max.: 615 nm) was used to stain dead cells. Images were captured using a TE 200‐E fluorescence microscope (Nikon Instruments, Italy) through ACT‐1 and ACT‐2 software for DXM120F digital cameras and analysed with ImageJ software [42].

### 2.4 Cell Cycle Assay

The impact of RSV on the cell cycle was analysed using 5‐bromodeoxyuridine (BrdU) and PI staining, followed by flow cytometry analysis (*n* = 3). The experiment and subsequent analysis were carried out following the methods of Romani et al. [44] and Esposito et al. [45]. OS cells were incubated for 1.5 h at 37°C with BrdU, a thymidine analogue, incorporated into newly synthesised DNA during the S phase but not in the G0/G1 or G2/M phases. The incorporated BrdU was detected using a primary anti‐BrdU antibody (BioLegend, San Diego, CA, USA) and revealed using a FITC conjugated secondary antibody (Cytek Biosciences, Fremont, CA, USA). Finally, cells were stained with PI (50 μg/mL, Sigma‐Aldrich). Analysis was performed using the FACS Calibur flow cytometer (BD Biosciences). Data analysis was performed using FloJo Software (Tree Star) [44, 45].

### 2.5 Cell Apoptosis Assay

The proapoptotic effects of RSV were assessed using the Apoptosis Kit—Annexin V Alexa Fluor 488 and Propidium Iodide (Life Technologies, Cat. no. A10788, Milan) (*n* = 3). The experiment and subsequent analysis were carried out following the methods of Romani et al. [44] and Esposito et al. [45]. OS cells (5 × 10^4^/well) were seeded in 24‐well plate and treated with RSV (100 μM) for 48 h. After treatment, cells were trypsinised, washed with 1× PBS and centrifuged at 8.000 RPM for 10 min. After discarding the supernatant, pellet was resuspended in 100 μL of Annexin Binding Buffer (ABB) 1X and incubated with 5 μL of Annexin‐V Alexa Fluor 488 and 1 μL of PI for 20 min Apoptotic cells exhibited green fluorescence (early apoptosis) or both green and red fluorescence (late apoptosis), while necrotic cells were only red. Analysis was performed with a FACS Calibur cytometer (BD Biosciences, San José, CA, USA) and FlowJo software (Tree Star, Ashland, OR, USA) [44, 45].

Apoptosis activation was also evaluated using the CellEvent Caspase‐3/7 Green Detection Reagent (Life Technologies, Milan, Italy, Cat. no. 10723) (*n* = 3). The experiment and subsequent analysis were carried out following the methods of Lanzilotti et al. [42]. This assay used a fluorogenic substrate specific for activated caspases‐3 and ‐7. Upon caspases activation in apoptotic cells, the substrate is cleaved, allowing the dye to bind DNA and generate a fluorogenic signal. OS cells (5 × 10^4^/well) were seeded in 24‐well plate were treated with RSV (100 μM), for 48 h. Cells were after stained with the CellEvent Reagent diluted in 1× PBS and incubated at 37°C for 30 min. Digital images were acquired using a TE 200‐E fluorescence microscope and captured with ACT‐1 and ACT‐2 software for DXM120F digital cameras (Nikon Instruments, Sesto Fiorentino, Italy) at 20× magnification. Image analysis was performed using ImageJ software [42].

### 2.6 Wound‐Healing Assay

To evaluate the effect of resveratrol on the migratory capacity of osteosarcoma (OS) cells, a wound‐healing assay was performed (*n* = 3). The experiment and subsequent analysis were carried out following the methods of Lanzilotti et al. [42]. OS cells (5 × 10^4^/well) were cultured in a 24‐well plate to 90% confluence, required for the wound‐healing assay, before a scratch was made with a pipette tip. Therefore, cells were treatment with RSV (100 μM) and image acquisition was performed at different time points 0, 24, 48, and 72 h (T0, T1, T2, T3) using a bright‐field microscope (TE2000E, Nikon S.p.A., Florence, Italy) at 4× magnification. Digital images were captured using ACT‐1 and ACT‐2 software for DXM1200F digital cameras (Nikon Instruments) [42].

Cell migration capacity was assessed by comparing the scratch areas before and after RSV treatment and analysed using ImageJ software. Results were expressed as the average percentage of wound closure (%) over a 72‐h period [42].

### 2.7 qRT‐PCR for Apoptotic Pathway and Human Extracellular Matrix and Adhesion Molecules

The *qRT‐PCR* Apoptosis Array allowed analysis the expression of 84 genes, including pro‐ and antiapoptotic pathways, death cell receptors and 5 housekeeping (HK) genes; the qRT‐PCR for human extracellular matrix and adhesion molecules allowed analysis the expression of 84 genes involved in cell‐to‐cell adhesion, cells to the ECM adhesion, and 5 HK genes. QuantiNova LNA PCR focus panel human apoptosis array (Qiagen, Milan, Italy, Cat. no. 249950) and the human extracellular matrix and adhesion molecules PCR array (Qiagen, Milan, Italy, Cat. no. 330231‐PAHS‐013ZA) were employed to analyse the genes expression in OS cells treated with RSV (100 μM) up to 48 h (*n* = 3). The experiment and subsequent analysis were carried out following the methods of Mazzoni et al. [46, 47]. Before qRT‐PCR Array, RNA was isolated using the RNeasy Plus mini Kit (Qiagen, Milan, Italy, Cat. no. 74104) following the provided protocol. RNA quality and quantity were evaluated using a Nanodrop spectrophotometer (ND‐1000, Nanodrop Technologies, Wilmington, DE, USA), then reverse transcribed to cDNA using QuantiNova Reverse Transcription Kit (Qiagen, Milan, Italy) for the Human Apoptosis PCR Array and the RT^2^ First Strand cDNA Kit (Qiagen, Milan, Italy) for The Human Extracellular Matrix and Adhesion Molecules Array [46, 47]. The Ribosomal Protein Lateral Stalk Subunit P0 (RPLP0) is the HK gene selected for data normalisation includes in qRT‐PCR Array plate. Analyses were carried out using CFX96 Touch PCR detection system (Bio‐Rad, Milan, Italy) [46, 47].

### 2.8 qRT‐PCR for Wnt/β‐Catenin Signalling Pathways

The expression of Wingless‐related integration site 1 (WNT1), c‐MYC and vimentin (VIM) genes, linked to Wnt/β‐catenin signalling, tumour progression and EMT mechanism was analysed via qRT‐PCR in RSV 100 μM–treated OS cells for 48 h (*n* = 3). RNA was extracted employing the RNeasy Plus Mini Kit (Qiagen, Milan, Italy). RNA quality and quantity was evaluated using a Nanodrop spectrophotometer. Total RNA was reverse transcribed into cDNA using the ImPromII Reverse Transcription System kit (Cat. no. A3800; Promega, Milan, Italy). Specific primers (forward and reverse) were used to amplify the genes of interest (HA1212981899 WT1 F, HA1212981900 WT1 R; HA12129848 VIM F, HA12129849 VIM R, Sigma‐Aldrich, Milan, Italy). Real‐Time PCR were performed using the Advanced Universal SYBR Green Supermix chemistry (Bio‐Rad, California, USA, Cat. no. 1,725,271). For data normalisation, the housekeeping gene encoding the 60S ribosomal protein L13a (RPL13A) (HA10668568 RPL13 F; HA10668569 RPL13 R, Sigma‐Aldrich, Milan, Italy) was chosen and amplified. Analyses were conducted using the CFX96 Touch PCR detection system (Bio‐Rad, Milan, Italy).

### 2.9 Vimentin and β‐Catenin Immunofluorescence

Immunofluorescence was performed to evaluate the expression of β‐Catenin and Vimentin proteins in OS cells (*n* = 3). The experiment and subsequent analysis were carried out following the methods of Mazzoni et al. [43, 46]. OS cells were seeded (2.5 × 10^4^ cells) on a 12 mm slide until 70% confluence and then treated with 100 μM RSV. After 48 h, slides were fixed in 4% paraformaldehyde (PFA) for 20 min (Sigma‐Aldrich, Milan, Italy) and permeabilised with 0.1% Triton X‐100 for 10 min at room temperature. Cells were incubated for 1 h at 37°C with the primary antibodies β‐catenin mouse monoclonal antibody (Thermo Fisher Scientific, Milan, Italy, Cat. no. 13–8400) and vimentin rabbit polyclonal antibody (Thermo Fisher Scientific, Milan, Italy, Cat n. PA5‐27231), diluted 1:150 and 1:100 in 1X PBS and 3% bovine serum albumin (BSA). Subsequently, cells were washed with 1X PBS and incubated for 1 h at 37°C with the secondary antibodies Alexa Fluor 488 IgG antirabbit (Thermo Fisher Scientific, Milan, Italy Cat. no. A‐11008) and Alexa Fluor 488 IgG antimouse (Thermo Fisher Scientific, Milan, Italy Cat n. A‐11001), diluted 1:400 and 1:250 in 1X PBS and 3% BSA. The secondary IgG antibody, targeting the primary antibody bound to vimentin, was diluted together with Phalloidin‐TRITC (Tetramethylrhodamine Isothiocyanate) (Sigma‐Aldrich, Milan, Italy), diluted 1:1000 in 1X PBS + 3% BSA, to stain actin filaments in red fluorescence. Nuclei were highlighted using 0.5 mg/mL DAPI (Sigma‐Aldrich, Milan, Italy). The images were acquired using a fluorescence microscope (TE2000E Nikon S.p.A., Florence, Italy) with 20× magnification and ACT‐1 and ACT‐2 software for DXM1200F digital cameras (Nikon Instruments, Florence, Italy) [43, 46].

### 2.10 Statistical Analysis

Statistical analysis of the experiments, performed in triplicate, was conducted using GraphPad Prism 9.0 software. Data were analysed using one‐way analysis of variance (ANOVA) and *t*‐tests, followed by Bonferroni's post hoc test, based on *n* = 3 independent experiments, each conducted in triplicate. *p* values < 0.05 were considered statistically significant. For the real‐time PCR, values were normalised to housekeeping genes, and the fold change (FC) for each gene expression was calculated using the 2^−ΔΔCT^ method. The FC values were plotted on a logarithmic scale (Log_2_ FC). A twofold up‐ or downregulated expression (Log_2_ FC > 1 or < −1) compared to controls was considered significant.

## 3 Results

### 3.1 Hyperspectral Imaging Reveals RSV Uptake in OS Cells

**FIGURE 1:**
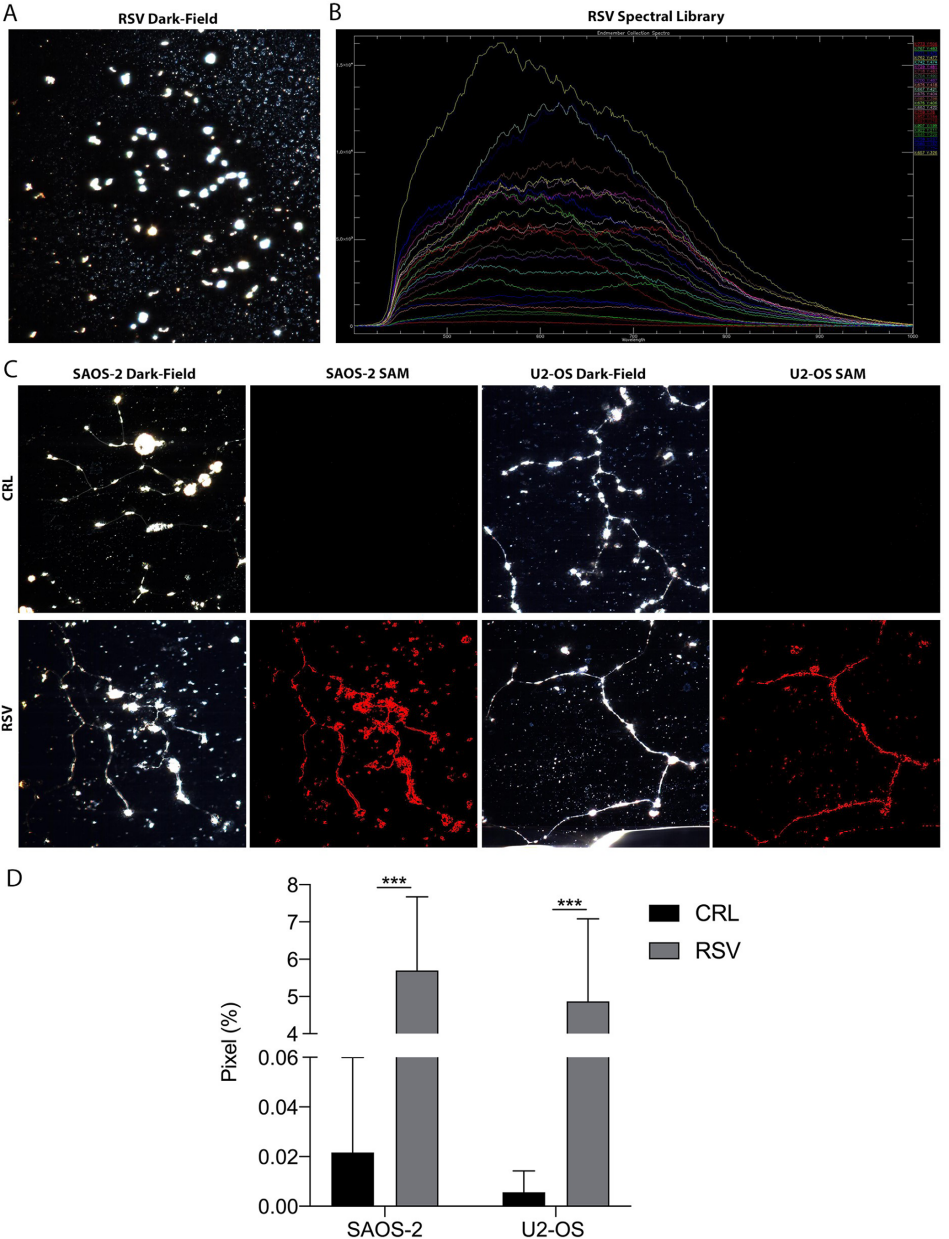
Hyperspectral Imaging (HSI) of RSV and intracellular localisation in both OS cell lines. (A) Dark‐field optical imaging captures the signal of pure RSV, and (B) the associated spectral library shows the intensity of scattered light across wavelengths. (C) Dark‐field images and Spectral Angle Mapping (SAM) analysis in OS cell lines (SAOS‐2 and U2‐OS) show no RSV‐associated signals in control samples (completely black images), while treated cells display coloured pixels matching the RSV spectral signature, confirming intracellular localisation of the compound. Imaging was performed using a 60× oil immersion objective to resolve RSV distribution and spectral characteristics. (D) Quantification of RSV‐positive pixels reveals a statistically significant increase in both treated OS cell lines, compared to controls (***p < 0.001), confirming cellular uptake of RSV.

Hyperspectral Imaging analysis allowed the evaluation of the uptake and localisation of the RSV compound within OS cells. The analysis detected C‐H and C = O bond vibrations, which differ in pure resveratrol compared with the entire cellular architecture. Thanks to this, the SAM algorithm was able to recognise the resveratrol spectra only in treated cells, indicating the compound's presence exclusively in these cells and providing proof of its entry into the cells. Figure 1 shows the dark‐field image of pure resveratrol (Figure 1A) and the corresponding spectral library (Figure 1B). The spectral graph displays the scattered light intensity versus the analysed wavelengths (λ). Figure 1 also presents a panel for both OS cell lines (Figure 1C), where dark‐field optical images and the SAM analysis results of control and treated cells are shown. The black images (where no resveratrol spectra were identified) confirm the absence of resveratrol in control cells. Conversely, the presence of resveratrol in treated cells is confirmed by coloured pixels, which correspond to the resveratrol spectra detected by SAM. The statistical analysis (Figure 1D) of the percentage of pixels occupied by resveratrol after SAM detection shows a significant increase in treated cells compared with the control (***p < 0.001), with a detected RSV percentage difference of 5.67% in SAOS‐2 and 4.86% in U2‐OS between control and treated samples.

### 3.2 RSV Activities on Cellular Proliferation and Viability

**FIGURE 2:**
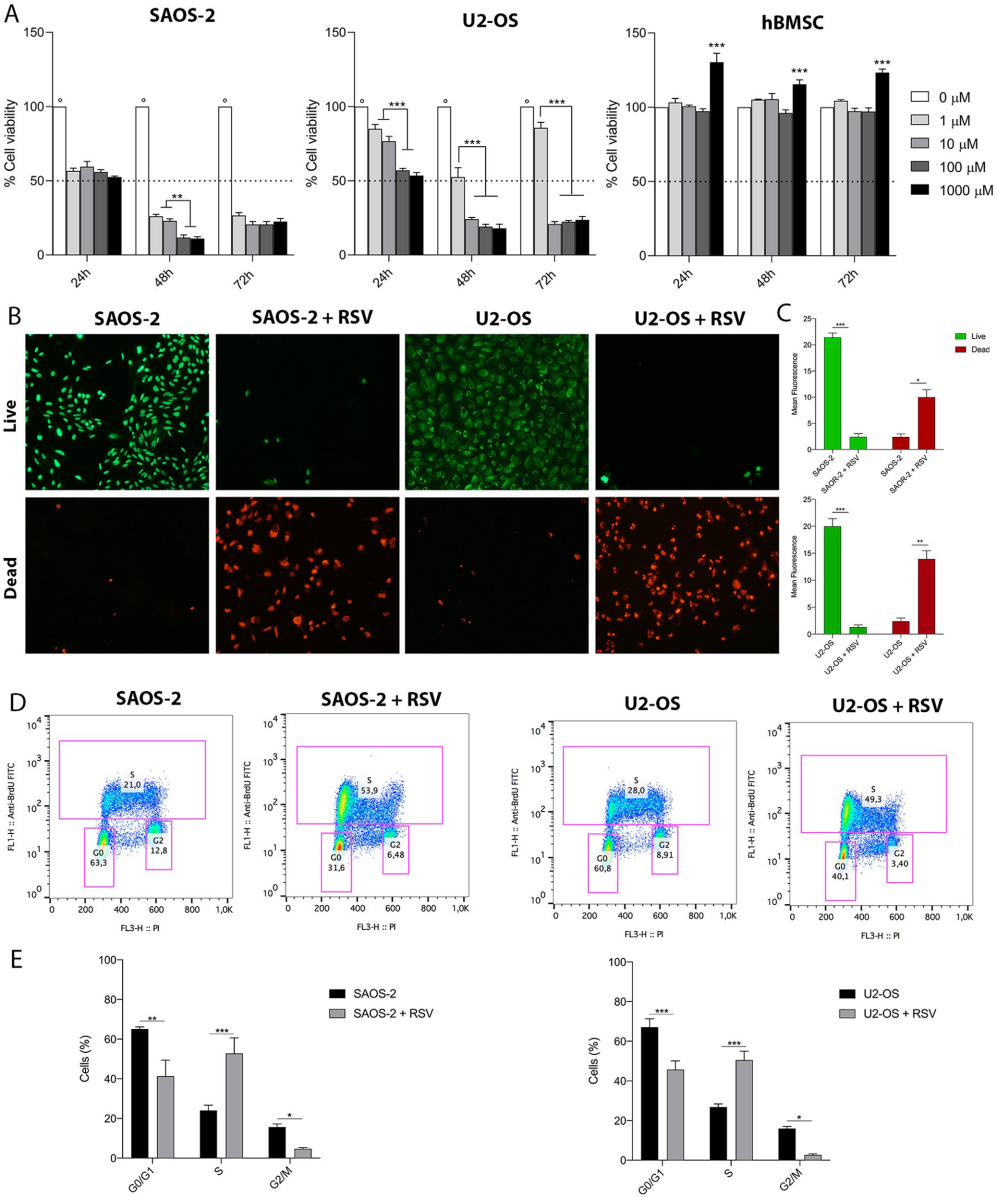
Effects of RSV on the proliferation, viability and cell cycle distribution of OS cell lines and hBMSCs. (A) The MTT assay was used to assess the effect of resveratrol (RSV, 1–1000 μM) on OS cell lines (SAOS‐2 and U2‐OS) and healthy hBMSCs over 24, 48 and 72 h. In SAOS‐2 cells, RSV reduced proliferation in a dose‐dependent manner, compared to control (*p* < 0.001), with significant decreases at 100 and 1000 μM after 48 h, compared to other treatments (**p < 0.01). In U2‐OS cells, RSV significantly reduced viability at all concentrations, with more differences observed after 24 h at higher concentrations (***p < 0.0001). hBMSCs showed no significant changes, except for a notable increase at 1000 μM (***p < 0.0001). (B) The Live/Dead assay confirmed the cytotoxic effect of RSV (100 μM) on OS cells after 48 h, using green Cyto‐dye for live cells and red propidium iodide for dead cells. (C) Fluorescence image quantification showed a significant reduction in live cells (***p < 0.0001) and an increase in dead cells (**p < 0.001; *p < 0.01) in RSV‐treated OS cell lines compared to the control. (D) The effect of RSV on the cell cycle was analysed by BrdU/PI staining and flow cytometry after 48 h of treatment with 100 μM. The cytogram displays that RSV treatment resulted in a significant accumulation of OS cells in the S phase and a decrease in the G0/G1 and G2/M phases, compared to untreated controls. (E) Statistical analysis confirmed a significant increase in the S phase (***p < 0.0001) and a significant reduction in the G0/G1 phase (*p < 0.001; ***p < 0.0001) in both OS cell lines.

MTT assay was performed to assess the effect of RSV on OS cell lines and on the healthy cell model hBMSCs. Cells were treated with different concentrations of RSV (1–1.000 μM) for 24, 48 and 72 h. RSV inhibited SAOS‐2 proliferation as follows: RSV 1 μM (57%–30%), RSV 10 μM (60%–22%), RSV 100 μM (56%–20%) and RSV 1000 μM (53%–22%) during the incubation of 24–72 h. In SAOS‐2, all treatments at all time points resulted in a significant reduction in viability compared to untreated controls, set at 100% (°*p* < 0.001). After 48 h, treatments at 100 and 1000 μM showed a significant reduction compared with other treatments (**p < 0.01) (Figure 2A). In U2‐OS cells, results showed reduction of cell proliferation as follows: RSV at 1 μM (86%–86%), RSV 10 μM (77%–22%), RSV 100 μM (57%–21%), and RSV 1000 μM (53%–23%) during the incubation of 24–72 h. U2‐OS treated showed a significant reduction in viability at all time points compared to untreated controls (°*p* < 0.001). After 24 h, 100 and 1000 μM treatments showed a significant reduction compared to other treatments (***p < 0.0001). At 48 and 72 h, 10, 100 and 1000 μM treatment showed a significant reduction in viability compared to the 1 μM (***p < 0.0001) (Figure 2A). RSV 1–1.000 μM was also employed to treat hBMSCs, demonstrating that the compound did not affect hBMSC proliferation, which maintained their viability across all time points and concentrations used. A statistically significant increase in cell growth was observed in the 1000 μM treatment (***p < 0.0001) (Figure 2A). RSV was able to impair cell proliferation, in both OS cell lines, in a dose‐dependent manner, at all the time points investigated. Based on these data, the concentration of 100 μM was selected to perform the subsequent analysis that were conducted at 48 h post‐treatment.

Live and dead analysis confirm the cytotoxic effects of RSV (100 μM) on OS cells at 48 h. Cyto‐dye and IP were used to stain live and dead cells, respectively (Figure 2B). Our data demonstrated that RSV was able to reduce OS cell viability, inducing cell death after 48 h of incubation. Fluorescence quantification of digital images showed a statistically significant reduction in live cells (***p < 0.0001) and a statistically significant increase in dead cells (**p < 0.001; *p < 0.01) in both OS cell lines treated compared to control (Figure 2C).

### 3.3 RSV Blocks Cell Cycle of OS Cells in S‐Phase

The distribution of OS cells in the different phases of the cell cycle was analysed using BrdU/PI staining in cultures treated with 100 μM RSV for 48 h, followed by flow cytometry analysis. Cells in the S‐phase are BrdU‐positive, while cells in the G0/G1 and G2/M phases are PI‐positive. The obtained data were processed and analysed using FlowJo software, which generated the cytogram (Figure 2D). Data are expressed as the percentage of the total population in each phase. The results show that SAOS‐2 exhibited a 32.9% increase in the S‐phase and a decrease in the G0/G1 and G2/M phases by 31.7% and 6.32%, respectively, after 48 h of treatment compared to control. U2‐OS showed a 21.3% increase in the S‐phase and a reduction in the G0/G1 and G2/M phases by 20.7% and 5.51%, respectively, after 48 h of treatment compared to control. Results show a significant increase of both OS cell lines in the S‐phase compared with the untreated control (***p < 0.0001) and a significant reduction of cells in the G0/G1 phase (**p < 0.001; ***p < 0.0001) (Figure 2E). Resveratrol induces cell cycle arrest and accumulation in the S‐phase, with a corresponding decrease of cells in the G0/G1 and G2/M phases.

### 3.4 RSV Induces the Apoptotic Process in OS Cell Lines

**FIGURE 3:**
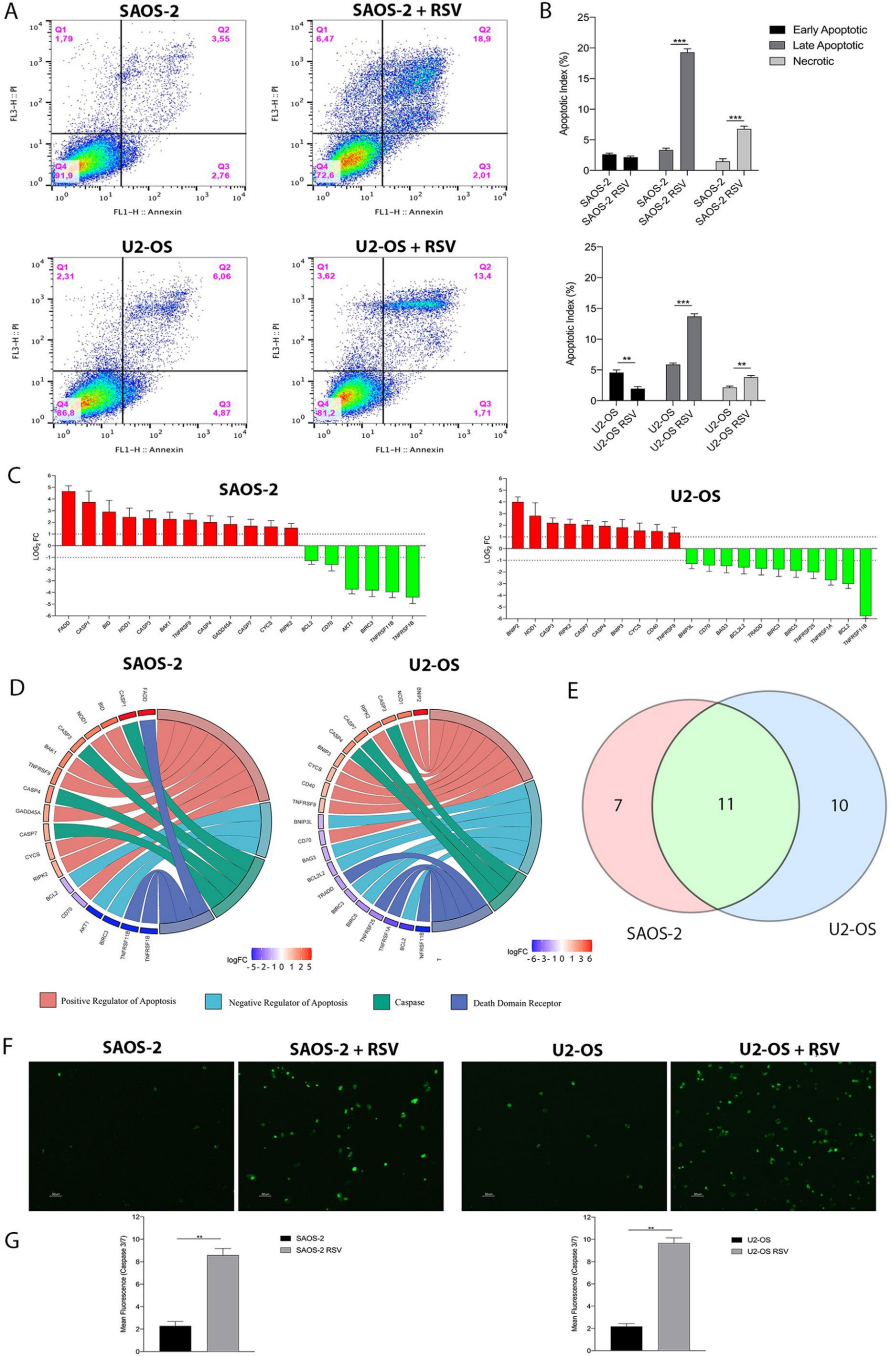
RSV induces apoptosis in OS cell lines. (A) Flow cytometry with Annexin V/PI staining was used to assess apoptosis in OS cells treated with 100 μM RSV for 48 h. The analysis identified early apoptotic (Annexin V+), late apoptotic (Annexin V+/PI+) and necrotic (PI+) cells, with Annexin V and PI intensities plotted on the X and Y axes, respectively. (B) Quantification showed that RSV significantly increased late apoptotic and necrotic cells in both OS cell lines (*p < 0.0001 for SAOS‐2 and **p < 0.01 for U2‐OS), compared to control. A decrease in early apoptotic cells is observed in U2‐OS (**p < 0.01). (C) Gene expression profiling after RSV treatment revealed 18 differentially expressed apoptotic genes in SAOS‐2 (12 upregulated, 6 downregulated) and 21 in U2‐OS (10 upregulated, 11 downregulated), based on a Log_2_ FC > 1 or < −1. (D) Gene Set Enrichment Analysis (GSEA) grouped these modulated genes into four categories: Positive regulators of apoptosis, negative regulators, caspases and death domain receptors, showing both up‐ and downregulated genes in each cell line. (E) A Venn diagram showed 11 apoptotic genes commonly modulated in both OS cell lines, with 7 unique to SAOS‐2 and 9 to U2‐OS, suggesting shared and cell‐specific mechanisms of RSV‐induced apoptosis. (F) Immunostaining for caspase‐3/7 revealed increased activation in both RSV‐treated OS cell lines after 48 h. (G) Fluorescence quantification using ImageJ confirmed a significant rise in activated caspase‐3/7 levels (**p < 0.001) in both RSV‐treated OS cells compared to controls.

Annexin V/Propidium Iodide staining was performed to assess RSV‐induced apoptosis and necrosis in OS cells after 48 h of treatment with 100 μM RSV. Early apoptotic cells were Annexin V‐positive, whereas necrotic cells were PI‐positive. Late apoptotic cells were positive for both stains. The samples were analysed by flow cytometry, and the obtained data were processed and analysed using FlowJo software, which generated a cytogram (Figure 3A).

In untreated SAOS‐2 cells, the percentage of live cells was 91.9%, while apoptotic and necrotic cells accounted for 8.1% (2.76% in early apoptosis, 1.79% in late apoptosis, and 3.55% in necrosis). In SAOS‐2 cells treated with 100 μM RSV, the percentage of live cells decreased to 72.6%, while apoptotic and necrotic cells increased to 27.38% (2.01% in early apoptosis, 18.9% in late apoptosis, and 6.47% in necrosis). In untreated U2‐OS cells, the percentage of live cells was 86.8%, while apoptotic and necrotic cells accounted for 13.24% (4.87% in early apoptosis, 6.06% in late apoptosis, and 2.31% in necrosis). In U2‐OS cells treated with 100 μM resveratrol, the percentage of live cells was 81.2%, while apoptotic and necrotic cells increased to 18.73% (1.71% in early apoptosis, 13.4% in late apoptosis, and 3.61% in necrosis). The graphs (Figure 3B) show a statistically significant increase in the percentage of late apoptotic and necrotic cells in SAOS‐2 treated with RSV, compared to control (***p < 0.0001). In U2‐OS cells treated with RSV, the graphs show a statistically significant increase in the percentage of late apoptotic and necrotic cells compared to control (***p < 0.0001; **p < 0.01). It is also detectable significant reduction in early apoptotic cells compared with the untreated U2‐OS (**p < 0.001).

### 3.5 RSV Modulates Apoptotic Gene Expression in OS Cell Lines

The Real‐Time PCR QuantiNova LNA PCR Focus Panel Human Apoptosis was used to evaluate the expression of 84 genes involved in Apoptotic process. Gene expression analysis in treated SAOS‐2 showed differential expression of 18 genes compared to control, 12 upregulated and 6 downregulated (Figure 3C, Table 1). In U2‐OS the differential expression of 21 genes was detected, of which 10 were upregulated and 11 were downregulated (Figure 3C, Table 2).

**TABLE 1:** List of genes of apoptosis found to be *upregulated and downregulated* in SAOS‐2 treated with RSV at 48 h.

Upregulated genes			Downregulated genes		
Number	Symbol	Fold‐Change	Number	Symbol	Fold‐Change
(Log_2_ FC)			(Log_2_ FC)		
1	*FADD*	4.32	1	*BCL2*	−4.06
2	*CASP1*	3.07	2	*CD70*	−3.64
3	*BID*	2.21	3	*SKT1*	−3.47
4	*NOD1*	1.91	4	*BIRK3*	−3.45
5	*CASP3*	1.88	5	*TNFRSF11B*	−1.25
6	*BAK1*	1.86	6	*TNFRSF1B*	−1.09
7	*TNFRSF9*	1.85			
8	*CASP4*	1.64			
9	*GADD45A*	1.38			
10	*CASP7*	1.30			
11	*CYCS*	1.28			
12	*RIPK2*	1.25			

*Note:* SAOS‐2 treated with RSV at 48 h shows upregulation of 12 genes (Log_2_ FC > 1) compared to control: Fas‐Associated death domain (*FADD*), caspase1 (*CASP‐1*), BH3 Interacting domain death agonist (*BID*), nucleotide‐binding oligomerization domain containing 1 (*NOD1*), caspase‐3 (*CASP‐3*), BCL‐2 antagonist killer 1 (*BAK1*), tumour necrosis factor receptor superfamily member 9 (*TNFRSF9*), caspase 4 (*CASP4*), growth arrest and DNA damage‐inducible alpha (*GADD45A*), caspase7 (*CASP‐7*), cytochrome C, somatic (*CYCS*), receptor‐interacting serine/threonine‐protein kinase 2 (*RIPK2*). Downregulated genes (Log2 FC < −1) in SAOS‐2 treated with RSV at 48 h are 6: Tumour necrosis factor receptor superfamily member 1B (*TNFRSF1B*), tumour necrosis factor receptor superfamily member 11B (*TNFRSF11B*), baculoviral IAP repeat containing 3 (*BIRC3*), AKT serine/threonine kinase 1 (*AKT1*), cluster of differentiation 70 (*CD70*), B‐cell lymphoma‐2 (*BCL‐2*).

**TABLE 2:** List of genes of apoptosis found to be *upregulated and downregulated* in U2‐OS treated with RSV at 48 h.

Upregulated genes			Downregulated genes		
Number	Symbol	Fold‐Change	Number	Symbol	Fold‐Change
(Log_2_ FC)			(Log_2_ FC)		
1	*BNIP2*	3.70	1	*TNFRSF11B*	−2.64
2	*NOD1*	2.03	2	*BCL2*	−2.63
3	*CASP3*	1.90	3	*TNFRSF1A*	−2.56
4	*RIPK2*	1.83	4	*TNFRSF25*	−2.40
5	*CASP7*	1.78	5	*BIRC5*	−1.89
6	*CASP4*	1.67	6	*BIRC3*	−1.87
7	*BNIP3*	1.34	7	*TRADD*	−1.84
8	*CYCS*	1.09	8	*BCL2L*	−1.82
9	*CD40*	1.07	9	*BAG3*	−1.74
10	*TNFRSF9*	1.04	10	*CD70*	−1.47
			11	*BNIP3L*	−1.32

*Note:* U2‐OS treated with RSV at 48 h shows upregulation of 10 genes (Log_2_ FC> 1) compared to control: BCL‐2 interacting protein 2 (*BNIP2*), nucleotide‐binding oligomerization domain containing 1 (*NOD1*), caspase‐3 (*CASP‐3*), receptor‐interacting serine/threonine‐protein kinase 2 (*RIPK2*), caspase‐7 (*CASP‐7*), caspase‐4 (*CASP‐Log_2_4*), BCL‐2 interacting protein 3 (*BNIP3*), cytochrome C, somatic (*CYCS*), cluster of differentiation 40 (*CD40*), tumour necrosis factor receptor superfamily member 9 (*TNFRSF9*). Downregulated genes (Log_2_ FC < −1) in U2‐OS treated with RSV at 48 h are 11: Tumour necrosis factor receptor superfamily member 11B (*TNFRSF11B*), tumour necrosis factor receptor superfamily member 1A (*TNFRSF1A*), tumour necrosis factor receptor superfamily member 25 (*TNFRSF25*), baculoviral IAP repeat containing 5 (*BIRC5*), baculoviral IAP repeat containing 3 (*BIRC3*), TNF receptor associated death domain (*TRADD*), BCL‐2 like 2 (*BCL2L2*), BCL‐2 associated athanogene 3 (*BAG3*), cluster of differentiation 70 (*CD70*), BCL‐2 interacting protein 3 like (*BNIP3L*).

A detailed analysis, performed through Gene Set Enrichment Analysis (GSEA) using KEGG software, allowed the grouping of modulated genes based on their molecular activity into four distinct categories: (i) Positive Regulator of Apoptosis; (ii) Negative Regulator of Apoptosis; (iii) Caspase and (iv) Death Domain Receptor (Figure 3D).

In SAOS‐2 treated with RSV, the upregulated genes belonging to the category of positive regulators of apoptosis are BH3 interacting domain death agonist (BID), nucleotide‐binding oligomerisation domain containing 1 (NOD1), BCL2 Antagonist/Killer 1 (BAK1), tumour necrosis factor receptor superfamily member 9 (TNFRSF9), growth arrest and DNA damage inducible alpha (GADD45A), cytochrome C, somatic (CYCS), and receptor interacting serine/threonine kinase 2 (RIPK2). In U2‐OS treated with RSV, the upregulated positive regulators of apoptosis genes are BCL2/adenovirus E1B 19 kDa interacting protein 2 (BNIP2), nucleotide‐binding oligomerisation domain containing 1 (NOD1), receptor‐interacting serine/threonine kinase 2 (RIPK2), BCL2/adenovirus E1B 19 kDa interacting protein 3 (BNIP3), cytochrome C, somatic (CYCS), CD40 molecule (CD40) and tumour necrosis factor receptor superfamily member 9 (TNFRSF9). In both OS cell lines, the gene cluster of differentiation 70 (CD70) is the only downregulated gene in the category of apoptosis positive regulators. In the category of apoptosis negative regulators, the analysis revealed only downregulated genes in both OS cell lines treated with RSV. In particular in SAOS‐2 the downregulated genes are B‐cell lymphoma 2 (BCL2), RAC‐alpha serine/threonine‐protein kinase (AKT1) and baculoviral IAP Repeat Containing 3 (BIRC3); while in U2‐OS the down regulated genes are BCL2/adenovirus E1B 19 kDa interacting protein 3‐like (BNIP3L), BCL2‐associated athanogene 3 (BAG3), BCL2‐like 2 (BCL2L2), baculoviral IAP Repeat Containing 3 (BIRC3), Baculoviral IAP Repeat Containing 5 (BIRC5) and B‐cell lymphoma 2 (BCL2). Caspase 3 (CASP3), caspase 7 (CASP7) and caspase 4 (CASP4) are found to be upregulated in both OS cell lines treated with RSV. In SAOS‐2 there is also an upregulation of caspase 1 (CASP1).

Among genes encoding for death domain receptors, in SAOS‐2 Fas‐associated death domain protein (FADD) was found to be upregulated, while tumour necrosis factor receptor superfamily member 1B (TNFRSF1B) was downregulated; in U2‐OS, TNF receptor‐associated death domain (TRADD), tumour necrosis factor receptor superfamily member 25 (TNFRSF25) and tumour necrosis factor receptor superfamily member 1A (TNFRSF1A) appear to be downregulated. The gene tumour necrosis factor receptor superfamily member 11B (TNFRSF11B) is downregulated in both OS cell lines treated with RSV. The Venn diagram illustrates the distribution of genes expressed in the two osteosarcoma cell lines, SAOS‐2 and U2‐OS. The analysis highlights 11 genes that are commonly modulated in both OS cell lines: NOD1, CASP3, CASP4, CASP7, RIPK2, CYCS, TNFRSF9, CD70, BCL2, BIRC3 and TNFRSF11B. The genes exclusively modulated in SAOS‐2 are 7: FADD, CASP1, BID, BAK1, GADD45A, AKT1 and TNFRSF1B, while in U2‐OS, nine genes are exclusively modulated: BNIP2, BNIP3, BNIP3L, CD40, BAG3, BCL2L2, TRADD, BIRC5 and TNFRSF25. The modulation of 11 genes common to both OS cell lines, mainly associated with apoptotic and inflammatory pathways, suggests that both cell types share fundamental mechanisms of stress response and cell death regulation following RSV treatment (Figure 3E, Table 3).

**TABLE 3:** List of common genes of apoptosis found to be modulated in both OS cell lines treated with RSV at 48 h.

Number	Symbol	SAOS‐2 Fold‐Change (Log_2_ FC)	U2‐OS Fold‐Change (Log_2_ FC)
Upregulated genes			
1	*NOD1*	1.91	2.03
2	*CASP3*	1.88	1.90
3	*RIPK2*	1.25	1.83
4	*CASP7*	1.30	1.78
5	*CASP4*	1.64	1.67
6	*CYCS*	1.28	1.09
7	*TNFRSF9*	1.85	1.04
Downregulated genes			
8	*CD70*	−3.64	−1.47
9	*BIRC3*	−3.45	−1.87
10	*BCL2*	−4.06	−2.63
11	*TNFRSF11B*	−1.25	−2.64

*Note:* In both OS cell lines, RSV modulated the expression of 11 common genes of Apoptosis. The genes upregulated in both OS cell lines are: Nucleotide‐binding oligomerization domain containing 1 (*NOD1*), caspase 3 (*CASP3*), receptor‐interacting serine/threonine‐protein kinase 2 (*RIPK2*), caspase 7 (*CASP7*), caspase 4 (*CASP4*), cytochrome C, somatic (*CYCS*) and tumour necrosis factor receptor superfamily member 9 (*TNFRSF9*). The genes downregulated in both OS cell lines are: cluster of differentiation 70 (*CD70*), baculoviral IAP repeat containing 3 (*BIRC3*), B‐cell lymphoma 2 (*BCL‐2*) and tumour necrosis factor receptor superfamily member 11B (*TNFRSF11B*).

To confirm the data obtained from the Real‐Time PCR, the protein expression of activated caspase 3 and caspase 7 was studied using an immunostaining assay in OS cells treated with RSV for 48 h. The caspase‐3/7‐specific fluorochrome revealed higher levels of caspase‐3/7 positive cells (green) in both OS cell lines exposed to RSV compared to controls (Figure 3F). Fluorescence quantification of digital images showed a statistically significant increase (**p < 0.001) in activated caspase‐3/7 (Figure 3G) in RSV‐treated OS cells compared with the control.

### 3.6 RSV Inhibits Cell Migration in OS Cells

**FIGURE 4:**
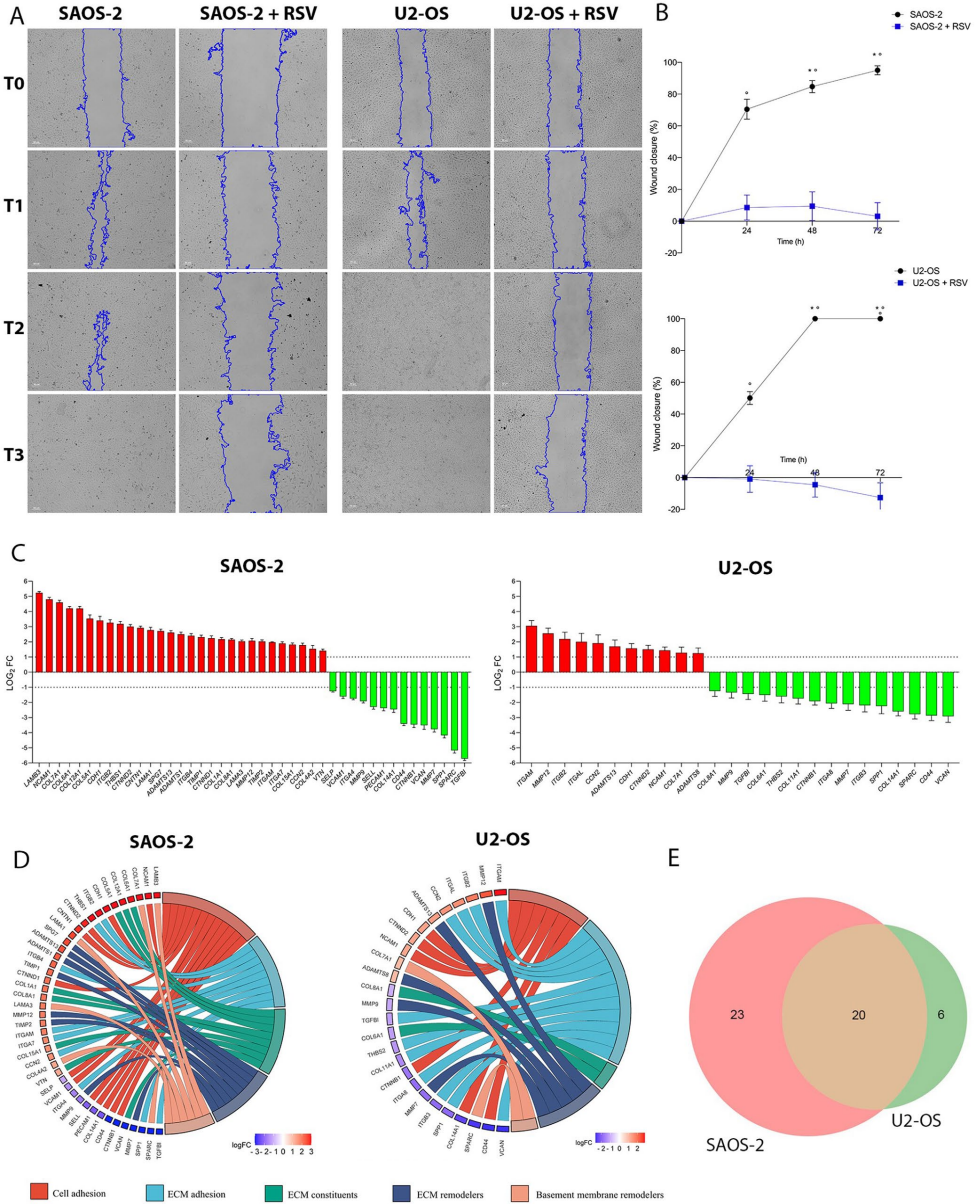
RSV inhibits cell migration and modulates ECM related gene expression in OS cells lines. (A) Bright‐field images from a wound healing assay show that control OS cells fully close the wound by 72 h (T3), whereas RSV‐treated cells (100 μM) exhibit no wound closure at any time point 0‐72 h (T0–T3), indicating that RSV strongly inhibits cell migration. (B) Quantitative analysis confirms significant wound closure in control cells over time compared to baseline T0 (0h) (°*p* < 0.0001), with additional increases at 48 h (T2) and 72 h (T3) compared to 24 h (T1) (*p < 0.001). Complete closure is observed at 72 h (T3) in control cells. (C) ECM‐related gene expression analysis using RT^2^ Profiler PCR Array shows differential expression in RSV‐treated cells: 43 genes are modulated in SAOS‐2 (29 upregulated, 14 downregulated) and 26 in U2‐OS (11 upregulated, 15 downregulated). (D) Gene Set Enrichment Analysis (GSEA) categorizes these genes into five functional groups: Cell–cell adhesion, ECM‐cell adhesion, ECM constituents, ECM remodelling and basement membrane components. Up‐ and downregulated genes are identified for each group in both cell lines. (E) A Venn diagram reveals 20 ECM‐related genes commonly modulated in both SAOS‐2 and U2‐OS, indicating shared pathways influenced by RSV, particularly those involved in cell adhesion and ECM remodelling.

The migration of OS cell lines was analysed to evaluate the anticancer potential of RSV 100 μM at 24, 48 and 72 h using a wound healing assay in OS cells treated with RSV 100 μM, compared to control. Images showed complete wound closure in untreated cells after 72 h, whereas in both OS cell lines treated, the wound maintains the same open area up to 72 h of treatment (Figure 4A). From microscope images, the wound area at different time points, was quantified. Subsequently, the percentage of wound closure was plotted for both control and treated OS cell lines at different time points 24, 48 and 72 h (T1, T2 and T3), relative to the wound area at T0, which was set at 0% (Figure 4B). Statistical analysis was performed by comparing the percentage of wound closure at the same time point between treated and untreated cells. The results show that both control cell lines exhibit significant wound closure at each time point compared to T0 (°*p* < 0.0001); additionally, wound closure at 48 h and 72 h is significant compared to 24 h (*p < 0.001). After 72 h of treatment, the control cells show complete wound closure (100%). Both treated cell lines show no significant differences in wound closure up to 72 h. These results confirm the ability of resveratrol to limit the cell migration process, significantly inhibiting the invasiveness of OS cells.

### 3.7 RSV Modulates ECM Expression Genes in OS Cell Lines

The RT^2^ Profiler PCR Array Human Extracellular Matrix & Adhesion Molecules kit was used to evaluate the expression of 84 genes involved in cell–cell and cell‐matrix interactions, as well as extracellular matrix remodelling. Gene expression analysis in treated SAOS‐2 showed differential expression of 43 genes compared to control, 29 upregulated and 14 downregulated (Figure 4C, Table 4). In U2‐OS the differential expression of 26 genes was detected, of which 11 were upregulated and 15 were downregulated (Figure 4C, Table 5). A detailed analysis performed through GSEA using KEGG software, allowed the grouping of modulated genes based on their molecular activity, into five distinct categories: (i) cell–cell adhesion genes; (ii) extracellular matrix (ECM) cell–matrix adhesion genes; (iii) extracellular matrix constituent genes; (iv) extracellular matrix remodelling genes; and (v) genes associated with the basement membrane (Figure 4D). In SAOS‐2 cells treated with resveratrol, the upregulated cell–cell adhesion genes include neural cell adhesion molecule 1 (NCAM1), cadherin 1 (CDH1), catenin delta 2 (CTNND2), contactin 1 (CNTN1) and catenin delta 1 (CTNND1), while the downregulated genes are P‐selectin (SELP), vascular cell adhesion molecule 1 (VCAM1), selectin L (SELL), platelet endothelial cell adhesion molecule 1 (PECAM1), collagen type XIV alpha 1 chain (COL14A1), cluster of differentiation 44 (CD44), and catenin beta 1 (CTNNB1). In U2‐OS cells, the upregulated adhesion genes are CDH1, CTNND2 and NCAM1, whereas CD44, COL14A1 and CTNNB1 are downregulated. For ECM‐cell adhesion, SAOS‐2 cells show upregulation of integrin beta 2 (ITGB2), thrombospondin 1 (THBS1), integrin beta 4 (ITGB4), integrin alpha M (ITGAM), integrin alpha 7 (ITGA7) and connective tissue growth factor (CCN2), while integrin alpha 4 (ITGA4), secreted phosphoprotein 1 (SPP1), and transforming growth factor beta‐induced (TGFBI) are downregulated. In U2‐OS, upregulated genes include ITGAM, ITGB2, integrin alpha L (ITGAL), and CCN2, while TGFBI, thrombospondin 2 (THBS2), collagen type XI alpha 1 chain (COL11A1), integrin alpha 8 (ITGA8), integrin beta 3 (ITGB3), SPP1 and versican (VCAN) are downregulated. Among ECM constituent genes, SAOS‐2 exhibits upregulation of collagen Type VI alpha 1 chain (COL6A1), collagen Type XII alpha 1 chain (COL12A1), collagen Type V alpha 1 chain (COL5A1), collagen Type I alpha 1 chain (COL1A1), collagen Type VIII alpha 1 chain (COL8A1), collagen Type XV alpha 1 chain (COL15A1) and vitronectin (VTN), whereas COL6A1 and COL8A1 are downregulated in U2‐OS. VCAN is downregulated in both cell lines. Regarding ECM remodelling, SAOS‐2 cells treated with RSV show upregulation of matrix metalloproteinase 12 (MMP12), spastic paraplegia 7 (SPG7), tissue inhibitor of metalloproteinase 1 (TIMP1) and tissue inhibitor of metalloproteinase 2 (TIMP2), while MMP7 and MMP9 are downregulated. In U2‐OS, MMP12 is upregulated, whereas MMP7 and MMP9 are downregulated. Among ADAMTS genes, SAOS‐2 shows upregulation of ADAMTS1, while U2‐OS shows upregulation of ADAMTS8. ADAMTS13 is upregulated in both cell lines. For basal membrane components, SAOS‐2 exhibits upregulation of laminin subunit alpha 1 (LAMA1), laminin subunit alpha 3 (LAMA3), laminin subunit beta 3 (LAMB3), collagen Type VII alpha 1 chain (COL7A1), and collagen Type IV alpha 2 chain (COL4A2), while secreted protein acidic and rich in cysteine (SPARC) is downregulated. In U2‐OS, COL7A1 is upregulated and SPARC is downregulated. Venn diagrams revealed that both cell lines share 20 modulated genes, indicating that resveratrol treatment affects similar biological pathways in both osteosarcoma models. Specifically, the regulation of genes related to cell adhesion and extracellular matrix dynamics may contribute to reducing the invasive potential and tumorigenic properties of osteosarcoma cells exposed to resveratrol (Figure 4E, Table 6).

**TABLE 4:** List of genes of extracellular matrix and adhesion molecules found to be *upregulated and downregulated* in SAOS‐2 treated with RSV at 48 h.

Upregulated genes			Downregulated genes		
Number	Symbol	Fold‐Change	Number	Symbol	Fold‐Change
(Log_2_ FC)			(Log_2_ FC)		
1	*LAMB*	5.18	1	*TGFBI*	−5.64
2	*NCAM1*	4.72	2	*SPARC*	−5.06
3	*COL7A1*	4.51	3	*SPP1*	−4.06
4	*COL6A1*	4.12	4	*MMP7*	−3.64
5	*COL12A1*	4.11	5	*VCAN*	−3.34
6	*COL5A1*	3.36	6	*CTNNB1*	−3.32
7	*CDH1*	3.20	7	*CD44*	−3.31
8	*ITGB2*	3.13	8	*COL14A1*	−2.29
9	*THBS1*	3.07	9	*PECAM1*	−2.25
10	*CTNND2*	2.90	10	*SELL*	−2.18
11	*CNTN1*	2.84	11	*MMP9*	−1.84
12	*LAMA1*	2.65	12	*ITGA4*	−1.69
13	*SPG7*	2.62	13	*VCAM1*	−1.51
14	*ADAMTS13*	2.52	14	*SELP*	−1.22
15	*ADAMTS1*	2.41			
16	*ITGB4*	2.30			
17	*TIMP1*	2.22			
18	*CTNND1*	2.13			
19	*COL1A1*	2.10			
20	*COL8A1*	2.09			
21	*LAMA3*	1.99			
22	*MMP12*	1.98			
23	*TIMP2*	1.97			
24	*ITGAM*	1.96			
25	*ITGA7*	1.82			
26	*COL15A1*	1.74			
27	*CCN2*	1.70			
28	*COL4A2*	1.38			
29	*VTN*	1.34			

*Note:* SAOS‐2 treated with RSV at 48 h shows upregulation of 29 genes (Log2 FC > 1) compared to control: Laminin Beta 3 (*LAMB3*), Neural Cell Adhesion Molecule 1 (*NCAM1*), Collagen Type VII Alpha 1 Chain (*COL7A1*), Collagen Type VI Alpha 1 Chain (*COL6A1*), Collagen Type XII Alpha 1 Chain (*COL12A1*), Collagen Type V Alpha 1 Chain (*COL5A1*), Cadherin 1 (*CDH1*), Integrin Subunit Beta 2 (*ITGB2*), Thrombospondin 1 (*THBS1*), Catenin Delta 2 (*CTNND2*), Contactin 1 (*CNTN1*), Laminin Alpha 1 (*LAMA1*), Spastic Paraplegia 7 (*SPG7*), A Disintegrin and Metalloproteinase with Thrombospondin Motifs 13 (*ADAMTS13*), A Disintegrin and Metalloproteinase with Thrombospondin Motifs 1 (*ADAMTS1*), Integrin Subunit Beta 4 (*ITGB4*), Tissue Inhibitor of Metalloproteinases 1 (*TIMP1*), Catenin Delta 1 (*CTNND1*), Collagen Type I Alpha 1 Chain (*COL1A1*), Collagen Type VIII Alpha 1 Chain (*COL8A1*), Laminin Alpha 3 (*LAMA3*), Matrix Metalloproteinase 12 (*MMP12*), Tissue Inhibitor of Metalloproteinases 2 (*TIMP2*), Integrin Alpha M (*ITGAM*), Integrin Alpha 7 (*ITGA7*), Collagen Type XV Alpha 1 Chain (*COL15A1*), Connective Tissue Growth Factor (*CCN2*), Collagen Type IV Alpha 2 Chain (*COL4A2*), Vitronectin (*VTN*). Downregulated genes (Log2 FC < −1) in SAOS‐2 treated with RSV at 48 h are 14: Transforming Growth Factor Beta‐Induced (*TGFBI*), Secreted Protein Acidic and Rich in Cysteine (*SPARC*), Secreted Phosphoprotein 1 (*SPP1*), Matrix Metalloproteinase 7 (*MMP7*), Versican (*VCAN*), Catenin Beta 1 (*CTNNB1*), Cluster of Differentiation 44 (*CD44*), Collagen Type XIV Alpha 1 Chain (*COL14A1*), Platelet Endothelial Cell Adhesion Molecule 1 (*PECAM1*), L‐Selectin (*SELL*), Matrix Metalloproteinase 9 (*MMP9*), Integrin Alpha 4 (*ITGA4*), Vascular Cell Adhesion Molecule 1 (*VCAM1*), P‐Selectin (*SELP*).

**TABLE 5:** List of genes of extracellular matrix and adhesion molecules found to be *upregulated and downregulated* in U2‐OS treated with RSV at 48 h.

Upregulated genes			Downregulated genes		
Number	Symbol	Fold‐Change	Number	Symbol	Fold‐Change
(Log_2_ FC)			(Log_2_ FC)		
1	*ITGAM*	2.81	1	*VCAN*	−2.64
2	*MMP12*	2.32	2	*CD44*	−2.63
3	*ITGB2*	1.87	3	*SPARC*	−2.56
4	*ITGAL*	1.62	4	*COL14A1*	−2.40
5	*CCN2*	1.54	5	*SPP1*	−1.89
6	*ADAMTS13*	1.40	6	*ITGB3*	−1.87
7	*CDH1*	1.35	7	*MMP7*	−1.84
8	*CTNND2*	1.31	8	*ITGA8*	−1.82
9	*NCAM1*	1.29	9	*CTNNB1*	−1.74
10	*COL7A1*	1.03	10	*COL11A1*	−1.47
11	*ADAMTS8*	1.01	11	*THBS2*	−1.32
			12	*COL6A2*	−1.22
			13	*TGFBI*	−1.18
			14	*MMP9*	−1.09
			15	*COL8A1*	−1.02

*Note:* U2‐OS treated with RSV at 48 h shows upregulation of 11 genes (Log_2_ FC > 1) compared to control: Integrin Subunit Alpha M (*ITGAM*), Matrix Metalloproteinase‐12 (*MMP12*), Integrin Subunit Beta 2 (*ITGB2*), Integrin Subunit Alpha L (*ITGAL*), Cellular Communication Network Factor 2 (*CCN2*), A Disintegrin and Metalloproteinase with Thrombospondin Type 1 Motif 13 (*ADAMTS13*), Cadherin 1 (*CDH1*), Catenin Delta 2 (*CTNND2*), Neural Cell Adhesion Molecule 1 (*NCAM1*), Collagen Type VII Alpha 1 Chain (*COL7A1*), A Disintegrin and Metalloproteinase with Thrombospondin Type 1 Motif 8 (*ADAMTS8*). Downregulated genes (Log2 FC < −1) in U2‐OS treated with RSV at 48 h are 15: Versican (*VCAN*), Cluster of Differentiation 44 (*CD44*), Secreted Protein Acidic and Rich in Cysteine (*SPARC*), Collagen Type XIV Alpha 1 Chain (*COL14A1*), Secreted Phosphoprotein 1 (*SPP1*), Integrin Subunit Beta 3 (*ITGB3*), Matrix Metalloproteinase‐7 (*MMP7*), Integrin Subunit Alpha 8 (*ITGA8*), Catenin Beta 1 (*CTNNB1*), Collagen Type XI Alpha 1 Chain (*COL11A1*), Thrombospondin 2 (*THBS2*), Collagen Type VI Alpha 1 Chain (*COL6A1*), Transforming Growth Factor Beta Induced (*TGFBI*), Matrix Metalloproteinase‐9 (*MMP9*), Collagen Type VIII Alpha 1 Chain (*COL8A1*).

**TABLE 6:** List of common genes of extracellular matrix and adhesion molecules found to be modulated in both OS cell lines treated with RSV at 48 h.

Number	Symbol	SAOS‐2 Fold‐Change (Log_2_ FC)	U2‐O2 Fold‐Change (Log_2_ FC)
Up‐regulated genes			
1	*ITGAM*	1.96	2.81
2	*MMP12*	1.98	2.31
3	*ITGB2*	3.13	1.87
4	*CCN2*	1.70	1.54
5	*ADAMTS13*	2.52	1.40
6	*CDH1*	3.20	1.35
7	*CTNND2*	2.90	1.31
8	*NCAM1*	4.72	1.29
9	*COL7A1*	4.51	1.03
10	*MMP9*	−1.84	−1.09
Down‐regulated genes			
11	*TGFBI*	−5.64	−1.18
12	*CTNNB1*	−3.32	−1.74
13	*MMP7*	−3.64	−1.84
14	*SPP1*	−4.06	−1.89
15	*COL14A1*	−2.32	−2.40
16	*SPARC*	−5.06	−2.56
17	*CD44*	−3.33	−2.64
18	*VCAN*	−3.32	−2.64
Oppositely regulated genes			
19	*COL8A1*	2.09	−1.02
20	*COL6A1*	4.12	−1.22

*Note:* In both OS cell lines, RSV modulated the expression of 20 common genes of extracellular matrix and adhesion molecules. The upregulated genes are: Integrin Subunit Alpha M *(ITGAM)*, Matrix Metallopeptidase 12 *(MMP12)*, Integrin Subunit Beta 2 *(ITGB2)*, Cellular Communication Network Factor 2 *(CCN2)*, A Disintegrin And Metalloproteinase With Thrombospondin Motifs 13 *(ADAMTS13)*, Cadherin 1 *(CDH1)*, Catenin Delta 2 *(CTNND2)*, Neural Cell Adhesion Molecule 1 *(NCAM1)*, Collagen Type VII Alpha 1 Chain *(COL7A1)*. The downregulated genes are: Matrix Metallopeptidase 9 *(MMP9)*, Transforming Growth Factor Beta Induced *(TGFBI)*, Catenin Beta 1 *(CTNNB1)*, Matrix Metallopeptidase 7 *(MMP7)*, Secreted Phosphoprotein 1 *(SPP1)*, Collagen Type XIV Alpha 1 Chain *(COL14A1)*, Secreted Protein Acidic and Cysteine Rich *(SPARC)*, CD44 Molecule (Indian Blood Group) *(CD44)*, Versican *(VCAN)*. Two genes are oppositely regulated, that is, upregulated in SAOS‐2 and downregulated in U2‐OS. These genes are: Collagen Type VIII Alpha 1 Chain *(COL8A1)*, Collagen Type VI Alpha 1 Chain *(COL6A1)*.

### 3.8 RSV Modulates the Wnt/β‐Catenin Signalling in OS Cells and Affects the Expression of Vimentin Protein, as Well as the Localisation of β‐Catenin Protein

**FIGURE 5:**
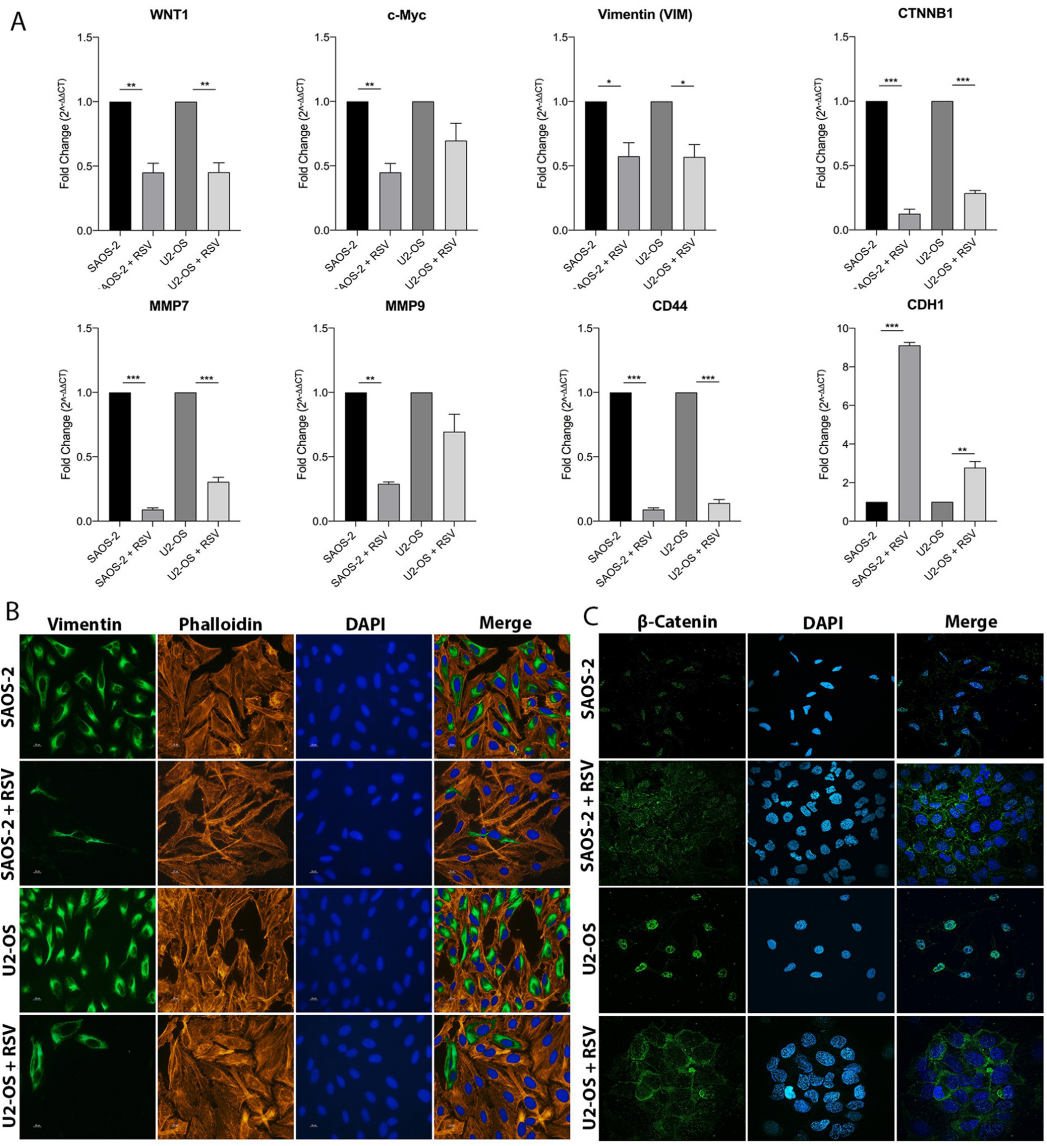
RSV modulates the Wnt/β‐Catenin signalling pathway and affects vimentin expression and β‐catenin localisation in OS cells lines. (A) Real‐time PCR analysis shows that RSV treatment (100 μM, 48 h) significantly downregulates key genes of the Wnt/β‐catenin pathway in SAOS‐2 and U2‐OS cells, including *CTNNB1*, *MMP7*, *MMP9* and *CD44* (*p < 0.001), all associated with ECM degradation, stemness and invasiveness. Conversely, *CDH1* (epithelial marker) is upregulated in both lines (*p < 0.001 for SAOS‐2 and **p < 0.01 for U2‐OS), suggesting a shift toward an epithelial phenotype. *WNT1* and *VIM* are significantly downregulated (*p < 0.01 and *p < 0.05, respectively) in both cell lines, with *c‐MYC* reduced in SAOS‐2 (*p < 0.01). (B) Immunocytochemistry reveals a notable decrease in Vimentin protein levels in RSV‐treated cells. Vimentin, a mesenchymal marker, appears less expressed, with treated cells showing morphological changes including elongated filaments and enlarged cell body and nucleus. (C) β‐catenin immunostaining indicates that RSV prevents its nuclear translocation, with the protein mainly localized at cell junctions and in the cytoplasm in treated cells, while in controls β‐catenin is predominantly nuclear, confirming RSV‐mediated inhibition of Wnt/β‐catenin signalling.

Some genes involved in the Wnt/β‐catenin pathway, implicated in the EMT mechanism, were significantly downregulated in the real‐time PCR Array analysis in both OS cells treated (Figure 5A). These genes include CTNNB1, encoding β‐catenin, which plays a role in cell adhesion and transcription of genes involved in tumour cell proliferation, migration and invasiveness (***p < 0.001); CD44, associated with stemness maintenance and cell invasiveness (***p < 0.001); MMP7, significantly down698 + 586regulated in both OS cell lines (***p < 0.001) and MMP9, significantly down‐regulated in SAOS‐2 (**p < 0.01), that are metalloprotease involved in extracellular matrix degradation and thus responsible for increasing the migratory and invasive capacity of tumour cells. Conversely, CDH1, an indicator of the epithelial phenotype, was upregulated in SAOS‐2 (***p < 0.001) and U2‐OS (**p < 0.01). Based on these results, further analysis was conducted to evaluate the expression profiles of other important genes that play distinct roles in Wnt/β‐catenin pathway, such as wingless‐type MMTV integration site family member 1 (WNT1), cellular myelocytomatosis Oncogene (c‐MYC), and Vimentin (VIM), using Real‐Time PCR in OS cells treated with 100 μM RSV after 48 h (Figure 5A). WNT1 is a ligand of the Wnt family that activates the Wnt/β‐catenin pathway. Its expression has been associated with increased cell invasiveness [48]. c‐MYC is a target gene of the Wnt/β‐catenin pathway. In the tumour context, its upregulation induced by β‐catenin, promotes proliferation and inhibits differentiation of tumour cells [49]. VIM is a cytoskeletal protein. The Wnt/β‐catenin pathway stimulates the expression of VIM, which promotes cell proliferation, metastasis and EMT in tumour cells [50]. The results of real‐time PCR show a statistically significant downregulation of WNT1 (**p < 0.01) and VIM (*p < 0.05) in both OS cell lines, compared to control, while c‐MYC appears downregulated with statistical significance in SAOS‐2 (*p < 0.01). The cytoskeletal architecture of OS cells was assessed through an immunocytochemistry assay. Specifically, the expression of vimentin was analysed using a green fluorescence antivimentin antibody to highlight intermediate filaments, while the distribution of actin filaments was examined using phalloidin‐TRITC, which emits red fluorescence. Vimentin is a protein abundantly expressed in cells with a mesenchymal phenotype, which are highly invasive and metastatic. It is overexpressed in certain tumours, including osteosarcoma [32]. No structural alterations were observed in actin filaments between treated and untreated groups. Vimentin protein expression showed a significant decrease in both OS cell lines treated with RSV compared to untreated controls. RSV also modified the cell morphology and cytoskeleton organisation, as evidenced by the more elongated appearance of Vimentin filaments and an expansion of both the cell body and nucleus in treated cells (Figure 5B). The effect of RSV on β‐catenin cellular localisation was evaluated with immunocytochemistry. Images show that RSV induced a significant inhibition of β‐catenin nuclear translocation in both OS cell lines, compared to control groups. In treated cells, β‐catenin remains localised at cell junctions and in the cytoplasm. Contrariwise, in the control groups, β‐catenin is predominantly found in the nucleus, indicating its active role as a transcription factor in the WNT pathway (Figure 5C).

## 4 Discussion

**FIGURE 6:**
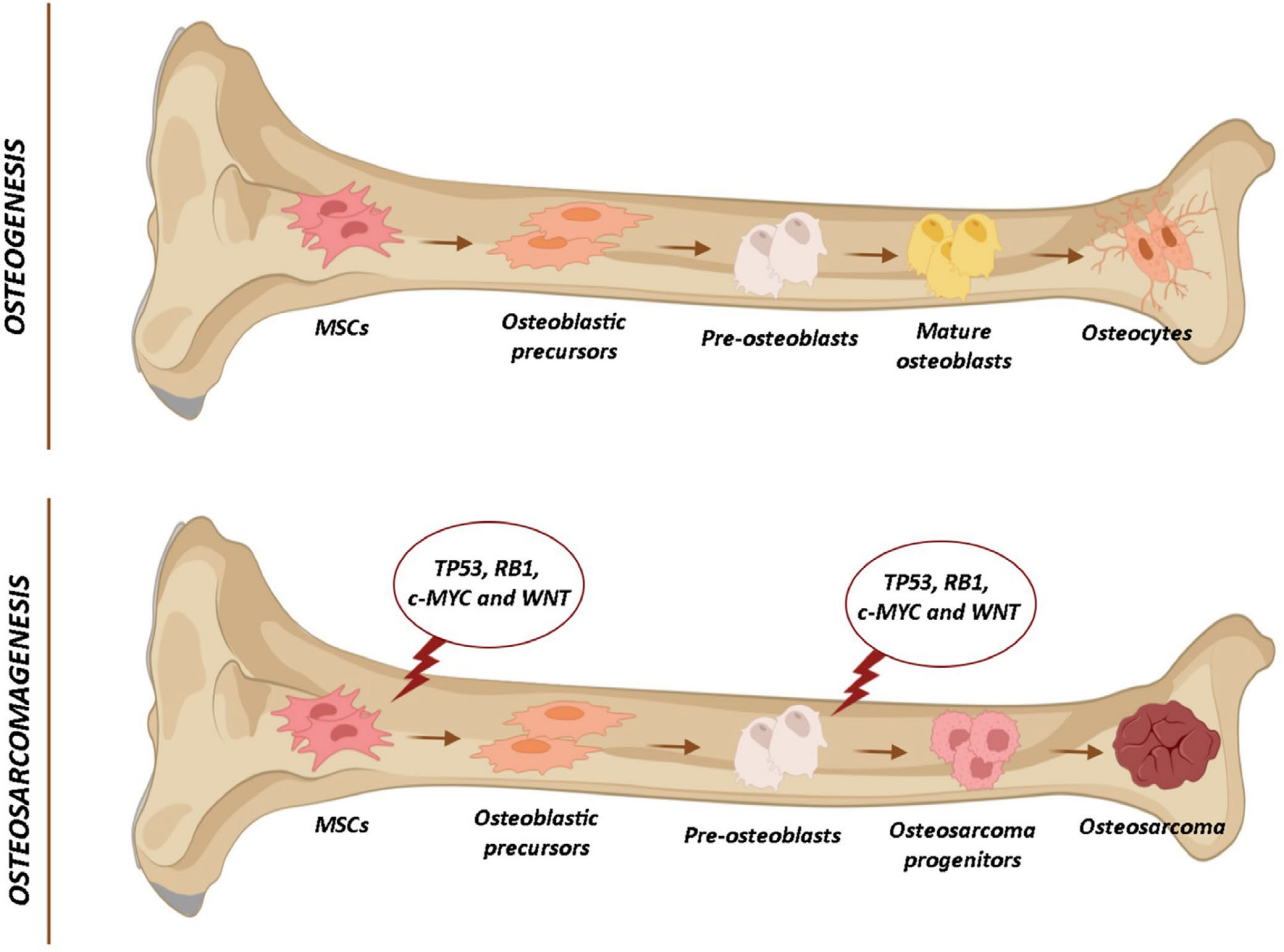
Molecular and cellular comparison between osteogenesis and osteosarcomagenesis. This Figure illustrates two distinct pathways: Osteogenesis and osteosarcomagenesis. In physiological bone formation, mesenchymal stem cells (MSCs) differentiate sequentially into osteoblastic precursors, preosteoblasts, mature osteoblasts and ultimately osteocytes, contributing to bone growth, remodelling and maintenance through a tightly regulated process. In contrast, osteosarcomagenesis is driven by genetic mutations in key regulatory genes (*TP53, RB1, c‐MYC, WNT*), which disrupt normal differentiation. These mutations prevent proper osteogenic maturation, leading MSCs to acquire tumorigenic potential. Impaired differentiation results in abnormal osteoblastic precursors with uncontrolled proliferation and further genetic instability. This leads to the formation of malignant, osteoblast‐like cells that eventually develop into aggressive osteosarcoma tumours. This comparison highlights the molecular mechanisms that distinguish physiological bone development from osteosarcomagenesis.

Osteosarcoma is the most common primary bone tumour, characterised by malignant mesenchymal cells that produce osteoid and immature bone [26]. It accounts for 8.9% of cancer‐related deaths and primarily affects children and adolescents [51]. OS develops due to genetic and epigenetic mutations in mesenchymal stem cells and osteoblastic progenitors. These changes disrupt osteogenesis, causing uncontrolled proliferation of partially differentiated osteoblasts and osteocytes, leading to osteosarcomagenesis [25]. Key mutations affect tumour suppressor genes like p53 and RB [27] and mutations in genes related to the Wnt/β‐catenin pathway [49] (Figure 6). Treatment typically combines chemotherapy and surgery to remove the primary tumour and metastases. However, chemotherapy has significant side effects, including chemoresistance, which remains a major challenge in OS management, emphasising the need for novel therapeutic strategies [26]. In recent years, natural compounds with potential antitumor effects have gained interest for their ability to enhance treatment efficacy while reducing the side effects of conventional chemotherapy. Among these, resveratrol—a polyphenolic compound found in various foods—has attracted significant scientific attention [52]. This study investigates the efficacy of RSV in inducing cytotoxic and proapoptotic effects in an in vitro model of OS cells (SAOS‐2 and U2‐OS). The research evaluates its impact on cell cycle arrest, inhibition of cell migration, and modulation of genes involved in cell–cell and cell–matrix adhesion mechanisms. Additionally, the study examines the effects of resveratrol on the expression of genes and proteins associated with epithelial–mesenchymal transition, tumour progression, metastasis and the Wnt/β‐catenin signalling pathway. The Dark‐Field‐HSI technology has already been used to discriminate between healthy and pathological tissues [53, 54, 55], to detect the presence of viruses [56, 57], as well as bacteria in plants [58]. In our study, HSI analysis was employed to localise RSV within treated OS cells. The SAM algorithm specifically identified resveratrol spectra in treated cells only, confirming the compound's exclusive presence in these cells and providing evidence of its cellular uptake. Moreover, resveratrol pixel localisation seems to correspond to both the nucleus and cytoplasm of the cells. The MTT assay demonstrated a dose‐ and time‐dependent inhibition of OS cell proliferation following RSV treatment, with the most significant effects observed at 48 and 72 h at concentrations of 100 and 1000 μM. Based on these findings, RSV was used at 100 μM for subsequent analyses, as this concentration reduced cell viability by 50% after 48 h, which was chosen as the time point for further experiments. Unlike its effects on OS cells, RSV did not exhibit cytotoxicity in hBMSCs, which maintained stable viability across all time points and concentrations. This selective cytotoxicity suggests that RSV can target malignant cells while preserving healthy bone tissue. These results align with previous studies indicating that RSV differentially affects tumour and healthy cells. In cancer cells, RSV downregulates antiapoptotic proteins while increasing proapoptotic factors, promoting caspase activation and DNA fragmentation, hallmarks of apoptosis. In contrast, in healthy cells, the balance between pro‐ and antiapoptotic proteins remains stable, preserving their functionality [24]. The Live/Dead assay confirmed RSV's cytotoxic effects on OS cells, showing a significant reduction in live cells and a corresponding increase in dead cells after 48 h of treatment with 100 μM RSV, supporting the hypothesis that RSV induces OS cell death. Cell cycle analysis revealed that RSV significantly altered cell cycle progression in OS cell lines. Flow cytometry using BrdU/PI staining demonstrated that 100 μM RSV treatment for 48 h resulted in a marked increase in S‐phase cells, with a corresponding decrease in G0/G1 and G2/M phases. These results align with previous studies on various human cancer cell lines (MCF7, SW480, HCE7, Seg‐1, Bic‐1 and HL60), where RSV induced S‐phase arrest by reducing the expression of key regulatory proteins such as cyclin D1, cyclin A, cyclin B1 and β‐catenin [59, 60]. This alteration appears to be mediated by proteasome activation, leading to the proteolytic degradation of these proteins, disrupting cell cycle progression and causing S‐phase arrest, thereby impairing DNA replication and mitotic progression [59]. Additionally, this study explored RSV's effects on apoptosis and necrosis in OS cell lines. The findings indicate that RSV treatment at 100 μM for 48 h significantly induces apoptosis in SAOS‐2 and U2‐OS cells. Flow cytometry analysis using Annexin V/PI staining revealed a substantial increase in apoptotic cells following RSV treatment compared to untreated controls. The gene expression analysis using real‐time PCR confirmed that RSV modulates key apoptotic factors in OS cell lines, activating genes involved in programmed cell death 48 h after treatment. SAOS‐2 exhibited differential expression of 18 genes, while U2‐OS showed 21 differentially expressed genes. Venn diagram analysis identified 11 commonly modulated apoptosis‐regulating genes, indicating that RSV influences shared apoptotic pathways in both osteosarcoma lines. This suggests that RSV induces apoptosis by activating caspases and inhibiting antiapoptotic factors, leading to a less aggressive tumour phenotype more responsive to treatment [61]. Among the upregulated proapoptotic genes in both cell lines, NOD1, TNFRSF9 and CYCS play crucial roles. NOD1 is a proapoptotic factor that increases cancer cells sensitivity to chemotherapy‐induced apoptosis, activating caspase‐3 and ‐9. Reduced NOD1 expression in certain tumours suggests it may act as a tumour suppressor [62, 63, 64]. TNFRSF9 is a co‐stimulatory receptor mainly expressed on activated T and NK cells. It enhances T and NK cell cytotoxicity against tumour cells and is being explored for immunotherapeutic applications [65]. CYCS encodes cytochrome c, a key apoptosis regulator. Loss of CYCS function can lead to tumorigenesis by allowing the accumulation of genetic mutations and uncontrolled cell division [66, 67]. In SAOS‐2 cells, BID, BAK1 and GADD45A were also upregulated. BID and BAK1 are both proapoptotic proteins and key members of the Bcl‐2 protein family. They contribute to mitochondrial permeabilisation, promoting the release of proapoptotic factors like cytochrome c, leading to caspase activation and cell death [68]. Both proteins downregulation or loss aids tumour progression and resistance to apoptosis [69, 70]. GADD45A plays a critical role in the G2/M checkpoint, preventing cell division with DNA damage [71]. Recognised as a tumour suppressor, GADD45A's presence influences disease progression in various tumoral conditions [72, 73, 74]. In U2‐OS, apoptosis activators BNIP2, BNIP3 and CD40 were upregulated. BNIP2 and BNIP3, belonging to the BNIP protein family, are involved in cell death regulation and autophagy. BNIP3 functions as a suppressor and also as a metastasis progression prognostic indicator of breast cancer [75]. BNIP2 regulates cell migration by activating Rho GTPase; its depletion in breast cancer cells increases migration and invasiveness by reducing RhoA activation [76]. CD40 can induce apoptosis in carcinoma cells through the activation of cytotoxic ligands of the TNF superfamily [77]. Furthermore, CD40 induced changes in MHC‐I, ICAM‐1 and Fas expression, enhancing the tumour's visibility to the immune system and promoting its elimination [78]. Conversely, the pro‐apoptotic gene CD70 was downregulated in both OS cell lines. It is overexpressed in various cancers, such as renal cell carcinoma, lymphoma and leukaemia. Due to its involvement in tumour progression, CD70 is a promising target for immunotherapy [79, 80].

Regarding the genes encoding cell death receptors, TNFRSF11B, also known as osteoprotegerin (OPG), is downregulated in both OS cell lines. In osteosarcoma, OPG regulates osteoclast activity, promoting bone resorption and may protect tumour cells from apoptosis, potentially enhancing tumour proliferation and bone metastasis [81]. TNFRSF25 and TNFRSF1A are downregulated in U2‐OS. These receptors, involved in immune regulation and apoptosis, activate prosurvival pathways such as NF‐kB and MAPK, implicated in oncogenesis and cancer progression [82, 83]. SAOS‐2 shows an upregulation of FADD, an adaptor protein that mediates apoptosis through receptors like Fas (CD95). Loss or dysfunction of FADD in tumour cells accelerates growth and metastasis [84, 85].

Among negative apoptosis regulators, BIRC3 was downregulated in both OS cell lines, while U2‐OS also exhibited BIRC5 downregulation. These genes inhibit caspase activation and their overexpression has been linked to cancer cell survival, tumour progression and chemoresistance [86]. BIRC3, specifically, is overexpressed in osteosarcoma cell lines, promoting tumour cell survival and progression [87]. BCL2 is downregulated in both OS cell lines, and U2‐OS also shows downregulation of BCL2L2. Both genes encode antiapoptotic proteins. A meta‐analysis revealed that positive BCL2 expression is associated with reduced 3‐year overall survival in OS patients [88]. Inhibiting BCL2 can restore doxorubicin sensitivity in resistant osteosarcoma cells, improving treatment efficacy [89]. Additionally, miR‐422a inhibits osteosarcoma cell proliferation by targeting BCL2L2, indicating that its inhibition could be a potential therapy for osteosarcoma [90]. SAOS‐2 treated with RSV also shows a downregulation of AKT1, a serine/threonine kinase that contributes to osteosarcoma tumorigenesis by activating the FOXO, PI3K‐Akt and MAPK signalling pathways, which are involved in tumour initiation and early metastasis [88, 89, 91].

SAOS‐2 show an upregulation of caspases 1, 3 and 7, while U2‐OS show an upregulation of caspases 3, 7 and 4. Caspases 1 and 4 are considered inflammatory caspases, that promoting cell death in response to stress factors such as inflammation and DNA damage [92, 93]. Their activation could contribute to the immune response in osteosarcoma. Caspases 3 and 7 are key executioner caspases of apoptosis. Specifically, in osteosarcoma, treatment sensitivity depends on caspase 3 and 7 activation. Chemotherapy‐resistant cells often exhibit impaired activation of these caspases [94]. According to gene expression analysis, immunostaining for activated caspase‐3/7 confirmed a significant increase in caspase‐3/7‐positive cells in both OS cell lines following RSV treatment, reinforcing its proapoptotic effect through executioner caspase activation. The migratory ability of OS cells following treatment with 100 μM RSV was assessed through the scratch test and the analysis of the expression of 84 genes involved in cell–cell and cell‐matrix interactions. The results of the scratch test demonstrate that resveratrol significantly inhibited the migration ability of the cells, compared to the control. Several scientific studies have reported that RSV inhibits the migration and invasion of tumour cells, mainly by acting on EMT and other pathways involved in metastasis [95]. Adhesion molecules are essential for maintaining epithelial integrity and regulating key cellular processes, such as cell shape, proliferation, differentiation and gene expression. Their dysregulation is linked to the EMT process, which promotes OS progression and metastasis [31, 36]. The study used Real‐Time PCR to analyse the expression of adhesion genes in OS cells treated with RSV. SAOS‐2 showed the modulation of 43 genes, while U2‐OS of 26 genes. Venn diagrams showed that 20 modulated genes are shared between the two cell lines, suggesting that RSV influences common biological processes in both OS cell lines, involved in cell adhesion and ECM processes, leading to a less invasive and tumorigenic phenotype in OS cells. A key finding was the down‐regulation of TGFBI, an ECM protein regulated by TGF‐β levels. TGFBI is implicated in tumour growth and drug resistance, and its knockdown inhibits invasion and migration in OS cells. Reduction of TGFBI expression may limit the tumorigenic processes induced by TGF‐β signalling [37, 96, 97]. The study also found a downregulation of cell–cell adhesion genes involved in the Wnt/β‐catenin signalling pathway, such as CD44 and CTNNBI. A meta‐analysis suggests that overexpression of β‐catenin in patients with OS, was associated with distant metastasis and poorer prognosis, suggesting that β‐catenin may be used as a prognostic biomarker of osteosarcoma [98]. CD44 has been identified as a positive regulator of the Wnt/β‐catenin pathway and can promote the proliferation and migration of OS cells. Additionally, CD44 has been proposed as OS prognostic biomarker for immune infiltration [99]. In contrast, CDH1 (encoding E‐cadherin) and NCAM1 were upregulated. E‐cadherin is crucial for maintaining tissue architecture, and its loss leads to EMT, increasing tumour invasiveness. Studies suggest that low E‐cadherin expression correlates with worse survival in OS [100]. NCAM1 plays a significant role in bone biology. It is expressed in osteoblasts and is involved in their differentiation [101]. Research indicates that NCAM1 downregulation is linked to poorer prognosis in malignancies [102, 103]. In SAOS‐2 the results indicate downregulation of cell–cell interaction selectins, particularly P‐selectin (SELP), expressed on activated platelets and endothelial cells, and L‐selectin (SELL), found on leukocytes. Cancer metastasis is facilitated by interactions between cancer cells and endothelial cells in distant tissues. Furthermore, interactions with platelets or leukocytes contribute to cancer cells extravasation and to the establishment of metastatic lesions. There are growing evidences that selectins contribute to cancer metastasis [104]. The integrin family consists of cell–ECM adhesion receptors that play a crucial role in tumour initiation, progression, and metastasis by mediating tumour cell proliferation, migration, and invasion. ITGAM and ITGB2 were upregulated in both cell lines. Studies suggest that ITGAM is positively correlated with macrophage polarisation from M0 to M1 and antitumor CD8+ T cells, inducing suppressive effect and better outcomes in OS patients [105]. ITGB2 has been shown to be an essential modulator of the immunological synapse between NK and a tumour cell and is therefore responsible for both adhesion and the targeted release of cytotoxic granules that kill the tumour cell. Therefore, the binding of a leukocyte through integrin β2 to a tumour cell can lead to tumour eradication [106]. ITGB4 and ITGA4 have been found to be modulated in the SAOS‐2 cell line. A previous study showed that higher expression levels of LAMB3 and ITGB4 predicted better overall survival rates and could be potential predictors of recurrent osteosarcoma prognosis. Both genes are upregulated in SAOS‐2 [107]. It has been shown that highly metastatic cancer cells express ITGA4 on the cell surface and adhere and migrate through VCAM‐1 [108]. Both ITGA4 and VCAM‐1 were found to be down expressed in SAOS‐2. ITGB3 has been found to be downmodulated in U2‐OS. ITGB3 is associated with tumorigenicity and cisplatin resistance in relapsed osteosarcoma, with its expression higher in recurrence tissues. Knockout of ITGB3 reduces osteosarcoma cell proliferation and migration, suggesting its role in tumour motility [109]. Trombospondins (THBS) are cell‐ECM adhesion glycoproteins and their dysregulation is associated with the development of various types of human malignant tumours and metastasis. THBS1 is upregulated in SAOS‐2 treated with resveratrol, while the U2‐OS exhibits a decrease of THBS2 expression. In OS, it was demonstrated that THBS1 is a potent inhibitor of the growth and metastasis of the osteosarcoma cell line MG‐63 [110]. THBS2 has been found to express at significantly high levels in patients with OS metastasis [111]. A study analysing the levels of THBS2 in osteosarcoma tumour tissues found that their expression was significantly higher compared to normal tissues [112]. Collagen is a major component of ECM and contributes significantly to bone's mechanical properties. Collagen plays a significant role in OS, impacting tumour development and progression. In our study, COL7A1 is found to be downregulated in both osteosarcoma cell lines. Evidence indicates that COL7A1 expression is often dysregulated in cancers and may serve as a prognostic biomarker, with higher expression linked to more aggressive disease and poorer outcomes [113]. Among genes encoding ECM constituent, a downregulation of VCAN in both SAOS‐2 and U2‐OS, was detected. VCAN is considered a key diagnostic gene and clinical predictor of OS progression. Studies have shown that VCAN expression are significantly higher in OS patients with worse outcomes, indicating its potential as early diagnosis marker and its relevance in cases with distant metastasis [114]. ECM remodelling plays a central role in osteosarcoma pathogenesis. If balance between ECM synthesis and degradation is disrupted, a pro‐aggressive tumour microenvironment is promoted [115]. MMPs are a group of proteolytic enzymes involved in ECM remodelling. Both MMP9 and MMP7 are transcriptional targets of the Wnt/β‐catenin signalling pathway, and this regulation plays a key role in promoting cancer cell invasion, migration and metastasis [116, 117]. These two proteins appear to be downregulated in both OS cell lines. MMP9 also enhanced tumour angiogenesis through the activation of proangiogenic factors such as VEGF [118]. MMP7 hyperactivity is associated with increased OS invasion of the vascular system and metastasis in lungs [119]. MMP12 was upregulated in both OS cells. Overexpression of MMP12 in OS leads to reduced tumour size, decreased angiogenesis, lower VEGF expression and increased expression of angiostatin. Mice with OS expressing high levels of MMP12 develop fewer metastases and have longer overall survival [120]. RSV treatment is therefore able to modulate numerous genes involved in cell adhesion, ECM composition and remodelling, tumour progression and invasion. Among these modulated genes, we find some important molecular targets and key components of the Wnt/β‐catenin pathway, such as CTNNB1, CD44, MMP7, MMP9 and CDH1, whose dysregulation in the oncological context is associated with the promotion of EMT. Our gene expression results are consistent with literature. Zou et al. [121] identified resveratrol as responsible for inhibiting the proliferation of human MG‐63 OS cells by downregulating the expression of β‐catenin. Xu et al. [122] also reported that polydatin, a glycoside derivative of resveratrol, can inhibit proliferation by suppressing β‐catenin signalling and induce apoptosis by increasing the Bax/Bcl‐2 ratio in OS cells. To complete the gene expression analysis, the expression levels of additional genes involved in Wnt/β‐catenin pathway were evaluated, namely WNT1, c‐MYC and VIM. The results showed a downregulation of these genes. The reduction of WNT1 expression leads to reduced activation of the Wnt/β‐catenin pathway and, therefore, of β‐catenin‐dependent oncogene transcription. Moreover, its downregulation can induce increased apoptosis, thus contributing to a reduction in tumour mass [123]. c‐MYC is an oncogenic transcription factor induced by β‐catenin, that stimulates tumour cell proliferation. High levels of c‐MYC are frequently associated with metastasis in OS patients and with poor prognosis. Recent studies have shown that the reduction of c‐MYC expression leads to decreased cell growth and increased apoptosis in OS cells in vitro [124]. The VIM gene encodes vimentin, a protein of the cytoskeleton that plays a crucial role in cell stability and motility. It is abundantly expressed in cells with a mesenchymal phenotype and represents a marker of EMT. Numerous studies demonstrate the overexpression of vimentin in human OS tumour tissue [125]. VIM downregulation of our study showed that RSV induces a reversal of the mesenchymal phenotype to an epithelial one, reducing the invasive and metastatic ability of OS cells. This result was confirmed by immunocytochemical analysis of vimentin protein expression 48 h after treatment. In untreated control, vimentin is highly expressed, whereas its expression significantly decreased in the treated cells. Immunocytochemistry images also showed that RSV induces a delocalisation of β‐catenin from the nucleus to the cell membrane in both OS cells. At the cell membrane, β‐catenin is part of an E‐cadherin‐containing multicomponent adherens junction complex, which is essential for cell–cell interactions. When junctions are disrupted, β‐catenin is released from the complex and, if the Wnt pathway is active, translocates to the nucleus to act as a transcription factor for EMT‐related genes [36]. The shift of β‐catenin from the cytoplasm to the membrane suggests that β‐catenin loses its role as a transcriptional activator of the Wnt pathway and instead functions as a cell–cell adhesion molecule. This change is associated with the acquisition of epithelial phenotype and a reduction in their migratory capacity of OS cells [126].

## 5 Conclusions

In summary, our study highlights the potential of RSV as a therapeutic agent for OS, demonstrating its ability to induce significant cytotoxic and pro‐apoptotic effects in OS cell lines. RSV treatment led to cell cycle arrest in the S‐phase, modulation of key apoptotic genes, and a reduction in cell migration and invasion. Key genes involved in apoptosis, such as NOD1, TNFRSF9 and CYCS, were upregulated, while antiapoptotic genes like BCL2 were downregulated. Furthermore, RSV influenced the expression of adhesion molecules and genes associated with the Wnt/β‐catenin signalling pathway, including MMPs, CTNNB1, CD44, CDH1, WNT1 and VIM, contributing to the reversal of EMT and inhibiting cell migration. These findings suggest that RSV may enhance the efficacy of osteosarcoma treatment by targeting crucial molecular pathways, providing a promising adjunct to conventional chemotherapy. Moreover, the selective cytotoxicity of RSV toward OS cells, with minimal impact on healthy bone cells, underscores its potential for reducing the side effects of chemotherapy, making it a valuable candidate for further investigation as a therapeutic strategy in osteosarcoma. Despite the promising results of this study, certain limitations must be acknowledged. First, the research was conducted exclusively in vitro using two osteosarcoma cell lines, which may not fully reflect the complexity of tumour behaviour in vivo. Moreover, the study does not assess the potential synergistic effects with standard chemotherapeutic agents. Future studies should aim to evaluate the efficacy and safety of resveratrol in animal models and investigate its potential role in combination therapies. Additionally, further functional studies are needed to clarify how resveratrol modulates key pathways, such as Wnt/β‐catenin, within the context of the tumour microenvironment.

## Author Contributions

**Roberta Chiarelli:** methodology. **Elisa Mazzoni:** writing original draft, conceptualization, and funding acquisition. **Raffaella De Pace:** writing – original draft, methodology. **Maria Rosa Iaquinta:** methodology. **Cinzia Brenna:** methodology. **Fabio Casciano:** investigation. **Maria Giulia Dell'Aquila:** investigation.

## Funding

This work was supported by the University of Ferrara, Fondo di Ateneo per la Ricerca, FAR 2023‐2024 grants to Elisa Mazzoni and FIRD grant 2023 to Elisa Mazzoni.

## Conflicts of Interest

The authors declare no conflicts of interest.

## Data Availability

The data that support the findings of this study are available from the corresponding author upon reasonable request.
